# SAFit2 ameliorates paclitaxel-induced neuropathic pain by reducing spinal gliosis and elevating pro-resolving lipid mediators

**DOI:** 10.1186/s12974-023-02835-5

**Published:** 2023-06-24

**Authors:** Saskia Wedel, Lisa Hahnefeld, Yannick Schreiber, Christian Namendorf, Tim Heymann, Manfred Uhr, Mathias V. Schmidt, Natasja de Bruin, Felix Hausch, Dominique Thomas, Gerd Geisslinger, Marco Sisignano

**Affiliations:** 1 Institute of Clinical Pharmacology, Pharmazentrum Frankfurt/ZAFES, University Hospital, Goethe-University, 60590 Frankfurt am Main, Germany; 2 Fraunhofer Institute for Translational Medicine and Pharmacology ITMP, and Fraunhofer Cluster of Excellence for Immune Mediated Diseases CIMD, 60596 Frankfurt am Main, Germany; 3 Core Unit Analytics and Mass Spectrometry, Max Planck Institute of Psychiatry, 80804 Munich, Germany; 4 Department of Biochemistry, Technical University of Darmstadt, 64287 Darmstadt, Germany

**Keywords:** SAFit2, FKBP51, Neuropathic pain, Chemotherapy, Oxylipins

## Abstract

**Background:**

Chemotherapy-induced neuropathic pain (CIPN) describes a pathological pain state that occurs dose-dependently as a side effect and can limit or even impede an effective cancer therapy. Unfortunately, current treatment possibilities for CIPN are remarkably confined and mostly inadequate as CIPN therapeutics themselves consist of low effectiveness and may induce severe side effects, pointing out CIPN as pathological entity with an emerging need for novel treatment targets. Here, we investigated whether the novel and highly specific FKBP51 inhibitor SAFit2 reduces paclitaxel-induced neuropathic pain.

**Methods:**

In this study, we used a well-established multiple low-dose paclitaxel model to investigate analgesic and anti-inflammatory properties of SAFit2. For this purpose, the behavior of the mice was recorded over 14 days and the mouse tissue was then analyzed using biochemical methods.

**Results:**

Here, we show that SAFit2 is capable to reduce paclitaxel-induced mechanical hypersensitivity in mice. In addition, we detected that SAFit2 shifts lipid levels in nervous tissue toward an anti-inflammatory and pro-resolving lipid profile that counteracts peripheral sensitization after paclitaxel treatment. Furthermore, SAFit2 reduced the activation of astrocytes and microglia in the spinal cord as well as the levels of pain-mediating chemokines. Its treatment also increased anti-inflammatory cytokines levels in neuronal tissues, ultimately leading to a resolution of neuroinflammation.

**Conclusions:**

In summary, SAFit2 shows antihyperalgesic properties as it ameliorates paclitaxel-induced neuropathic pain by reducing peripheral sensitization and resolving neuroinflammation. Therefore, we consider SAFit2 as a potential novel drug candidate for the treatment of paclitaxel-induced neuropathic pain.

**Supplementary Information:**

The online version contains supplementary material available at 10.1186/s12974-023-02835-5.

## Background

Paclitaxel is a chemotherapeutic agent, which is used for the treatment of malignancies as it stabilizes microtubule formation and thereby disrupts cell proliferation and cytoskeleton related cellular functions [1, 2]. Based on this mechanism of action, paclitaxel became a very important cytostatic, which enables the treatment of many different tumor types. However, the microtubule-stabilizing property of paclitaxel also affects the axonal transport of sensory neurons in the peripheral nervous system, which leads to axonal degeneration, neuropathy and neuropathic pain [3].

Neuropathic pain is a pathophysiological pain state, which is generally induced by nerve lesions or damages in the somatosensory system and can become chronic [4, 5]. A specific type of neuropathic pain is the chemotherapy-induced peripheral neuropathy (CIPN), which arises during chemotherapy as a severe side effect [6]. CIPN is a dose-dependent side effect that can even limit or impede an effective cancer therapy as chemotherapy often has to be reduced in dose or frequency or interrupted after the occurrence of CIPN [7, 8]. Unfortunately, CIPN affects up to 80 percent of all cancer patients and the majority of paclitaxel-treated patients [3]. Nonetheless, the available treatment possibilities for CIPN are extremely confined and mostly inadequate as the therapeutics of CIPN itself consist of a low effectiveness and may induce severe side effects, pointing out CIPN as pathological entity with an emerging need for novel treatment targets [9]. In addition, CIPN comprises a large symptom scope, which needs to be further investigated and underlying mechanisms to be revealed in order to develop appropriate therapeutic drugs.

Interestingly, previous studies revealed the FK506 binding protein 51 (FKBP51, encoded by the FKBP5 gene) as a novel and potential target for several pathological disorders such as chronic pain [10, 11], stress endocrinology [12] and glucocorticoid signaling related diseases [13]. In the context of chronic pain, FKBP51 was shown to be significantly upregulated in sensory neurons of the dorsal horn in an ankle joint knee inflammation model, indicating its involvement in the development of persistent pain states [10]. Based on these previous findings, we synthesized a highly potent and FKBP51 specific inhibitor named SAFit2 to address the scarcity of therapeutic approaches for chronic and especially paclitaxel-mediated neuropathic pain [14, 15].

To investigate whether or not the novel and specific FKBP51 inhibitor SAFit2 may reduce paclitaxel-induced neuropathic pain and improve paclitaxel-induced dysregulations of endogenous mechanisms, we performed a multiple low-dose paclitaxel mouse model. Previous findings already discovered that paclitaxel mediates axonal degeneration and its cumulative toxicity induces neuroinflammation and spinal cord gliosis [3]. Here, we confirm that a paclitaxel treatment induces a release of proalgesic mediators in nervous tissue and mediates spinal cord gliosis. In addition, we show that SAFit2 counteracts spinal cord gliosis after paclitaxel treatment and ameliorates paclitaxel-induced mechanical hypersensitivity in vivo. Previous studies have already shown alterations in the distribution of lipids and the levels of lipid mediators in nerve disorders or chronic pain states [4, 16, 17]. However, the role and precise mechanisms of endogenous lipid mediators in the context of paclitaxel-mediated neuropathic pain are still unclear. Based on this lack of understanding, we performed an unbiased lipidomics screen (LC–HRMS) and a targeted LC–MS/MS oxylipin screen from dorsal root ganglia (DRGs) and spinal cord (SC) samples to elucidate changes in the distribution of lipids after paclitaxel treatment. Furthermore, we assessed the influence of SAFit2 on paclitaxel-mediated alterations in lipid levels. Investigating these lipid levels in paclitaxel-treated mice, we observed that a SAFit2 treatment leads to an increase of anti-inflammatory and pro-resolving lipid mediators, which play a crucial role in the resolution of pain states.

## Materials and methods

### Animals

For all experimental approaches, wild-type animals with a C57BL/NRj background were purchased from the breeding companies Janvier and Charles River. All animal experiments, which included treated animals, were started with nine weeks old mice and naïve mice were matched in age and sex, respectively.

### Paclitaxel and SAFit2 treatment

**Fig. 1:**
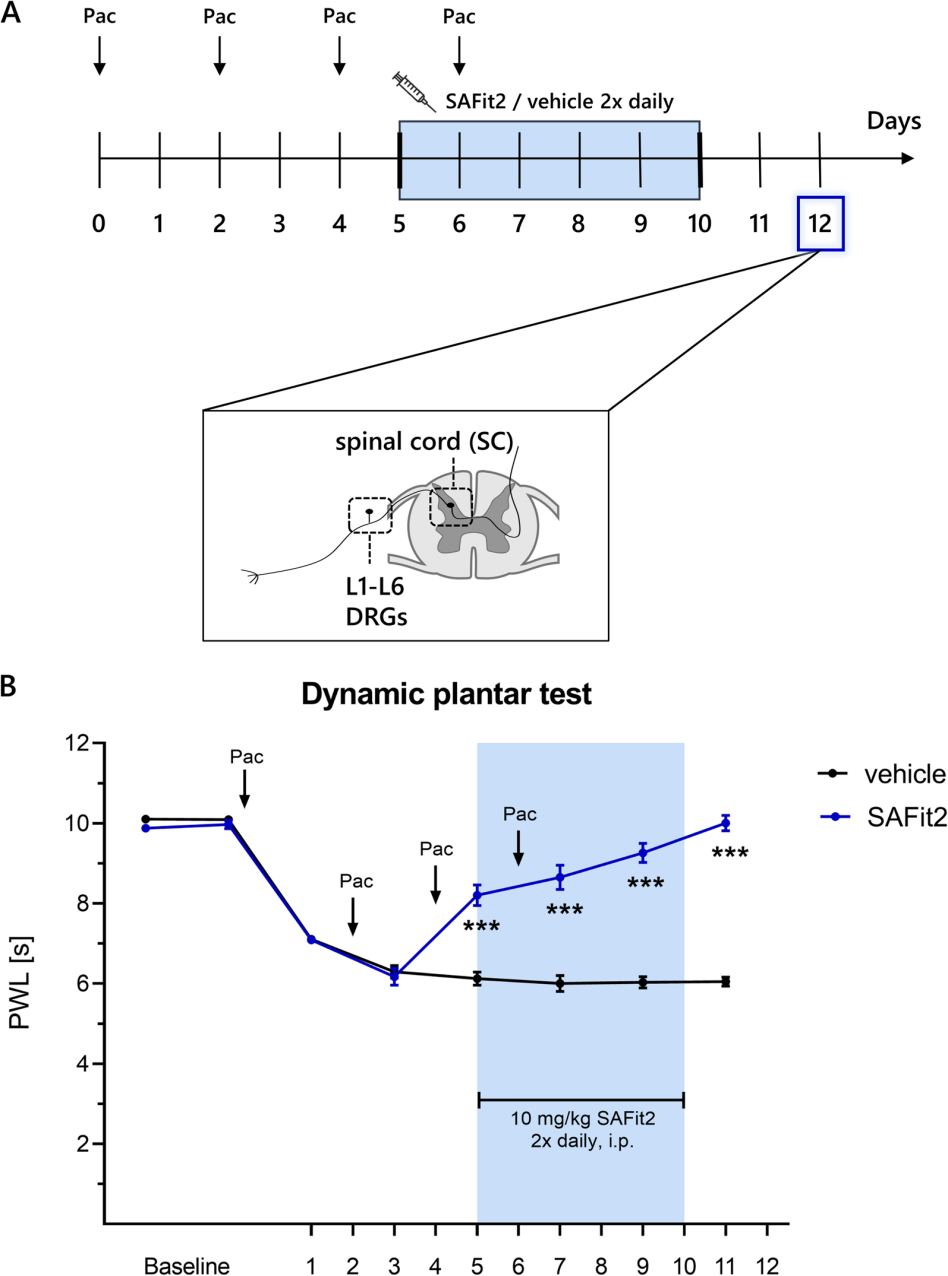
SAFit2 reduces paclitaxel-induced mechanical hypersensitivity. **A** Schematic illustration of the animal treatment: neuropathic pain was induced with a multiple low-dose paclitaxel model, including four intraperitoneal injections with 2 mg/kg paclitaxel on day zero, two, four and six. Afterwards, the animals were treated with 10 mg/kg SAFit2 intraperitoneally from day five to ten two times daily. At day twelve, the mice were killed and DRGs and spinal cord samples collected. **B** Baseline measurements were performed on two different days before paclitaxel treatment. The mechanical pain threshold was assessed over 12 days every second day and the behavioral readout within the SAFit2 treatment was recorded between the SAFit2 treatments, approximately three hours after the first dose of SAFi2. The data represent the mean ± SEM from 13–14 mice per group. ***p < 0.001 two-way ANOVA with Bonferroni´s post hoc test. *Pac* paclitaxel, *DRGs* dorsal root ganglia, *PWL* paw withdrawal latency, *SAFit2* selective antagonist of FKBP51 by induced fit 2

To induce paclitaxel-mediated neuropathic pain in mice, a multiple low-dose model was performed including four injections of 2 mg/kg paclitaxel, which was administered every second day, adding up to a cumulative dose of 8 mg/kg. [18]. The cytostatic paclitaxel was dissolved in a 50:50 (v/v) ethanol–cremophor EL (238470, Millipore) solution and stored at − 20 °C until further usage. Before the intraperitoneal injection, the paclitaxel stock-solution was thawed and freshly diluted 1:2 (v/v) with saline solution (NaCl 0.9% (w/v)) to a final concentration of 0.4 mg/ml. For assessing the influence of SAFit2 on paclitaxel-induced neuropathic pain and endogenous mechanisms, the mice were treated intraperitoneally with either 10 mg/kg SAFit2 or vehicle (PBS supplemented with 5% PEG400, 5% Tween and 0.7% ethanol) two times daily on six consecutive days, starting on day five after the first paclitaxel injection. The experimental treatment schedule is displayed in Fig. 1A.

### Behavioral experiments

Before starting the behavioral experiments and treatment phases, the locomotor function of all animals was verified using a rotarod assay [19]. During all behavioral experiments, the experimenter was blinded. For assessing the mechanical pain threshold, the animals were placed at least one hour before the measurement in the respective test cages to allow habituation. After this habituation phase, the mechanical paw withdrawal threshold was assessed using a Dynamic Plantar Aesthesiometer (Ugo Basile) as described previously [20]. The baseline measurements were performed on two consecutive days before the treatment phase and the mechanical pain thresholds were assessed every second day.

### Tissue isolation

For tissue isolation, mice were euthanized by isoflurane anesthesia (2–2.5% isoflurane in carbogen), cardio puncture, and cervical dislocation 12 days after the first paclitaxel injection. The lumbar (L1–L6) dorsal root ganglia (DRGs) and the respective innervated segments of the spinal cord were dissected followed by freezing tissue samples in liquid nitrogen for either RNA isolation, a multiplex assay or LC–HRMS and LC–MS/MS analysis. For immunohistochemistry staining, the spinal cord was dissected and fixed with 2% paraformaldehyde (PFA) solution until further usage.

### Pharmacokinetic study

In the pharmacokinetic study, a single dose of 40 mg/kg SAFit2, formulated as described above, was applied intraperitoneally. Likewise, a single dose of 100 mg/kg SAFit2, formulated as a slow-release formulation with vesicular phospholipid gel (VPG) [21], was applied subcutaneously. For the first formulation, plasma and brain samples were collected after 15 min, 30 min, 1 h, 3 h, 6 h and 24 h from three animals. For the second formulation, plasma and brain samples were collected after 2 h, 8 h, 24 h, 48 h, 72 h and 96 h from three animals. Mice were euthanized by isoflurane anesthesia (2–2.5% isoflurane in carbogen), cardiac puncture, and cervical dislocation. Brains were weighed and then homogenized in tenfold PBS volume containing Complete Protease Inhibitor Cocktail Tablets (Roche), using a Dispomix Drive (Medic Tools AG). Plasma samples were analyzed using the combined high-performance liquid chromatography/mass spectrometry (HPLC/MS–MS) technique. Analysis was performed using a Shimadzu Nexera X2 (Shimadzu) liquid chromatograph, which was interfaced to the ESI source of a Sciex QTrap 5500 (Sciex) triple quadrupole mass spectrometer. All samples were prepared using Ostro protein precipitation and phospholipid removal plates (Waters). Chromatography was accomplished using a gradient elution in an Accucore RP-MS column (100 × 2.1 mm, 2.6 µm Thermo Scientific) at a flow rate of 0.5 ml/min and 30 °C. The composition of eluent B was methanol with 10 mM ammonium formate with 0,1% formic acid and water with 10 mM ammonium formate with 0,1% formic acid was eluent A. The gradient was 0–3 min 60% B, 3–4.5 min 60–90% B, 0.5 min held at 90% B, 5–5.2 min 90–60% B and 5.2–6 min 60% B. The total run time was 6 min and the injection volume was 2 µl. The ion source was operated in the positive mode at 500 °C, and multiple reaction monitoring (MRM) collision-induced dissociation (CID) were performed using nitrogen gas as the collision gas. Deuterated SAFit2 (SAFit2-D3) was used as internal standard.

### Quantitative real-time PCR

To isolate the RNA from lumbar DRGs and the respective segments of the spinal cord, the mirVana miRNA Isolation Kit (Applied Biosystems) was used according to the manufacturer´s instructions. Afterwards, RNA concentrations were quantified with a NanoDrop ND-1000 spectrophotometer (NanoDrop Technologies) and a cDNA synthesis was conducted with 200 ng RNA from DRGs and 400 ng RNA from spinal cord. The reverse transcription was performed with the First Strand cDNA Synthesis Kit (Thermo Fisher Scientific) according to the manufacturer’s recommendations. The quantitative real-time PCR was performed with the QuantStudio™ Design & Analysis Software v 1.4.3 (Thermo Fisher Scientific) in a TaqMan® Gene Expression Assay System (Table 1, Thermo Fisher Scientific) according to the manufacturer´s instructions. The raw data were evaluated using the ΔΔC(T) method, as described previously [22, 23].

**Table 1:** List of used TaqMan® gene expression assays

Target	Gene	Article number	Company
ALOX12	Arachidonate 12-lipoxygenase	Mm00545833_m1	Thermo Fisher
ALOX15	Arachidonate 15-lipoxygenase	Mm00507789_m1	Thermo Fisher
ALOX5	Arachidonate 5-lipoxygenase	Mm01182747_m1	Thermo Fisher
ATF3	Activating transcription factor 3	Mm00476033_m1	Thermo Fisher
CerS5	Ceramide synthase 5	Mm00556165_m1	Thermo Fisher
CerS6	Ceramide synthase 6	Mm00510998_m1	Thermo Fisher
cFOS	FBJ osteosarcoma oncogene	Mm00487425_m1	Thermo Fisher
COX2	Cytochrome c oxidase subunit II	Mm03294838_g1	Thermo Fisher
CYP2J6	Cytochrome P450, family 2, subfamily j, polypeptide 6	Mm01268197_m1	Thermo Fisher
CYP3a11	Cytochrome P450, family 3, subfamily a, polypeptide 11	Mm00731567_m1	Thermo Fisher
GAPDH	Glyceraldehyde-3-phosphate dehydrogenase	Mm99999915_g1	Thermo Fisher
iNOS	Inducible nitric oxide synthase 2	Mm00440502_m1	Thermo Fisher
MMP9	Matrix metallopeptidase 9	Mm00442991_m1	Thermo Fisher
NOX2	NADPH oxidase 2	Mm01287743_m1	Thermo Fisher
NOX4	NADPH oxidase 4	Mm00479246_m1	Thermo Fisher
PLA2g4a	Phospholipase A2, group 4a	Mm00447040_m1	Thermo Fisher
PLA2g4c	Phospholipase A2, group 4c	Mm01195718_m1	Thermo Fisher
XDH	Xanthine dehydrogenase	Mm00442110_m1	Thermo Fisher

### Multiplex assay

For performing the ProcartaPlex multiplex immunoassay (Thermo Fisher), proteins were isolated with a manufacturer recommended cell lysis buffer, which was further supplemented with a phosphatase inhibitor cocktail (PhosSTOP, Roche) and a protease inhibitor cocktail (cOmplete, Roche). Each lumbar DRG (L1–L6 per animal) sample was suspended in 100 µl and each spinal cord sample, which comprises the respective innervated segments of the spinal cord, in 200 µl cell lysis buffer. The spinal cord samples were further processed by a cell grinder. Afterwards, the tissue was homogenized two times per sample using a Sonopuls Sonicator (Bandelin) with the setting 6 × 10%. During sonication, the samples were cooled in an ice bath, preventing proteins from degradation. Finally, the samples were centrifuged with 16,000 × *g* at 4 °C for 10 min, followed by collecting the supernatant for a protein concentration determination via Bradford.

The ProcartaPlex multiplex immunoassay was performed according to the manufacturer’s recommendations. Briefly, a dark wall 96-well plate was prepared by several washing steps and coating steps with respective magnetic beads. Afterwards, standards were prepared in a serial dilution (1:4, (v/v)) and added to the plate, followed by further washing steps. Lastly, the samples were diluted (1:2, (v/v)) and added to the plate, which was sealed and incubated for 40 min with 500 rpm on an orbital shaker at room temperature, overnight at 4 °C and further 50 min with 500 rpm at room temperature. On the next day, the detection antibody mixture was added after a washing step and the plate was further incubated on an orbital shaker for 30 min at room temperature. Again, the plate was washed and streptavidin phycoerythrin (SAPE) was added to the plate and incubated for 30 min as described above. After the last washing step, the plate was prepared with reading buffer, incubated for 5 min with 500 rpm on an orbital shaker, and measured with the Luminex 200 system (Bio-Rad).

### Immunohistochemistry

Before the immunohistochemical staining, the spinal cord samples were fixed with 2% PFA for 5 h at room temperature, as previously described in the tissue isolation section. As a next step, the tissue was dehydrated with a concentration series of 20% (w/v) and 30% (w/v) sucrose solutions. Therefore, spinal cord samples were firstly incubated in a 20% sucrose solution at 4 °C overnight and then further dehydrated in a 30% sucrose solution for five hours at 4 °C. Next, the spinal cord samples were embedded in Tissue-Tek and frozen at − 80 °C. The frozen tissue samples were serially sliced into 14-µm tissue slices with a cryostat (Leica Biosystems).

For staining the slices, the samples were fixed with 2% PFA for ten minutes. Afterwards, the fixative was removed and the slices rinsed with PBS. Next, slices were permeabilized with PBS (pH 7.4) containing 0.1% Triton X (v/v) for 15 min. After removing the PBST, the slices were blocked with a 3% BSA (w/v) solution for two hours at room temperature. The primary antibodies anti-GFAP (ab7260, Abcam), anti-IBA1 (019-19741, FUJIFILM Wako Pure Chemical Corporation), anti-ATF3 (sc-188, Santa Cruz) and anti-cFOS (9F3, Cell Signaling) were diluted 1:1000, 1:500, 1:100 and 1:200, respectively, in 1% BSA solution and applied for an overnight incubation at 4 °C. The primary antibody anti-FKBP51 (sc-271547, Santa Cruz) was diluted 1:50 as recommended in 3% BSA solution to minimize cross-reactivity as the host species is mouse.

On the next day, the excess of non-specific binding primary antibodies was removed via three washing steps with PBS, this time incubating the slices with PBS for 5 min. Afterwards, the secondary antibodies, goat anti-rabbit Alexa Fluor 488 (ab150077, Abcam) and donkey anti-rabbit Alexa Fluor 647 (A-31573, Thermo Fisher) were diluted 1:1000 in 1% BSA solution and applied for one hour at room temperature. The secondary antibodies goat anti-mouse Alexa Fluor 488 (CF488A, Biotium) and sheep anti-mouse Cy3 (C2181, Sigma Aldrich) were diluted in 3% BSA solution. Next, the excess of non-specific binding secondary antibodies was removed, the slices rinsed with PBS and further incubated with DAPI 1:1000 (6335.1, Carl Roth) for ten minutes at room temperature. The slices were mounted with Fluoromount-G mounting medium from Southern Biotech. Images were taken with the fluorescence microscope Observer.Z1 (Carl Zeiss) and processed as well as quantified with ImageJ software. Three to five representative images were taken per stained spinal cord slice, resulting in a total number of 10–20 images per animal. In total, we quantified the number of cells for the markers GFAP and IBA1 by counting cells in 70–80 pictures per condition. For the marker cFOS, we counted the cells in 40 images per condition and for FKBP51, we quantified the mean intensity per image for 40 images per condition.

### Liquid chromatography–high-resolution mass spectrometry (LC–HRMS)

For LC–HRMS purposes, an approximately 3 mg lumbar DRG sample and 30 mg of the respective parts of the spinal cord (SC) were homogenized using a pellet pestle mixer (Thermo Fisher scientific). The tissue samples were grinded for 30 to 60 s, with short breaks included to avoid overheating, in an ethanol:water (1:9, v/v) solution, using 119 µl ethanol for DRG samples and 1190 µl ethanol for spinal cord samples. Afterwards, lipid extraction and chromatographic separation was performed as well as the measurement settings and internal standards chosen as previously described [24]. Instrumental setup consisted of a Vanquish Horizon coupled to an Exploris 480 (both Thermo Fisher Scientific) with Zorbax RRHD Eclipse Plus C8 column (2.1 × 50 mm, 1.8 µm, Agilent) and a pre-column of the same type. The measured lipid levels were normalized to the respective protein amount of the sample. For all lipidomic measurements, including extraction and analysis, the experimenter was blinded.

### PLA_2_ activity assay

To measure PLA_2_ activity in primary sensory neuron lysates, DRGs were collected from two naïve mice and washed with ice-cold PBS. Next, the cells were lysed in 1 ml of ice-cold PBS and homogenized using a Sonopuls Sonicator (Bandelin) three times with the setting 6 × 10%. Afterwards, the homogenate was centrifuged with 10,000 rpm for 15 min at 4 °C and the supernatant collect for the PLA_2_ activity assay (Abcam). The assay was performed according the manufacturer’s recommendations.

### Oxylipin screen

For oxylipin analysis, dorsal root ganglia (DRG) were homogenized in 10% ethanol with addition of 10 M indomethacin (0.04 mg tissue/µl) with a pellet pestle mixer (fisher scientific). Similarly, spinal cord samples were homogenized in 10% ethanol with addition of 10 M indomethacin (0.075 mg tissue/µl) and 7 zirconium oxide beads using a Precellys 24-Dual tissue homogenizer coupled with a Cryolys cooling module (both Bertin Technologies) that was kept at < 10 °C. The Precellys program encompassed three rounds at 6500 × *g* for 20 s with 60 s pauses in between to avoid overheating. Tissue homogenates were stored on ice during extraction. For both tissue types, to 200 µl of tissue homogenate, 10 µl of 4.4% formic acid, 20 µl internal standard solution and 20 µl of 0.1% BHT in methanol were added and vortexed for 30 s. Proteins were precipitated by addition of 200 µl cold acetonitrile:methanol (8:2, v/v). After vortexing for 120 s and 600 s of centrifugation at approximately 15,000 × *g*, the supernatant was used for solid phase extraction using EVOLUTE EXPRESS ABN 30 mg/1 ml cartridges (Biotage) as previously described [25]. Calibration standards and quality control samples were prepared accordingly with 20 µl of standard solution in methanol with 0.1% BHT and 200 µL of PBS. Additionally, DRG homogenates were diluted by adding 180 µl of 10% ethanol with 10 M indomethacin to 20 µl of homogenate and processed as described above. LC–MS/MS analysis was then conducted on a QTrap 6500 + (Sciex) coupled to a 1290 Infinity II LC-System (Agilent) using an Acquity UPLC BEH C18 column (1.7 µm 100 × 2.1 mm ID) with a pre-column of the same type. For all measurements, including extraction and analysis, the experimenter was blinded. Further details including method parameters can be found in a previous publication [25].

### Data analysis and statistics

All data are presented as mean ± SEM. Normal distribution was confirmed using the Shapiro–Wilk test. The statistical analysis for behavioral experiments was conducted using a two-way analysis of variance (ANOVA) with Bonferroni’s post hoc correction. For all ex vivo experiments, an unpaired and heteroskedastic Student’s *t* test with Welch correction was performed, when comparing only two groups with each other. For the comparison of more than two groups, a one-way ANOVA was used and for examining the effect of two independent variables on a continuous dependent variable a two-way ANOVA was conducted. For all statistical analysis the software GraphPad Prism 9.5 was used. A *p* value of < 0.05 was considered as statistically significant.

## Results

Previous studies already suggested a crucial role of FKBP51 in the context of chronic pain states [10, 11]. However, its upregulation in chemotherapy-induced neuropathic pain is not confirmed yet. Therefore, we performed an immunohistochemistry staining to investigate its expression after paclitaxel treatment in L4–L5 dorsal root ganglia as well as in spinal cord slices (Additional file 1: Fig. S1). For both neuronal tissues, we observed a significant increase of FKBP51 after paclitaxel, when comparing the expression to naïve animals. These data confirm the relevance of FKBP51 in the context of chronic pain states and especially chemotherapy-induced neuropathic pain.

### SAFit2 ameliorates paclitaxel-induced mechanical hypersensitivity

To investigate the influence of SAFit2 on paclitaxel-induced neuropathic pain and underlying mechanisms, we chose a well-established multiple low-dose paclitaxel model as a multiple low-dose model corresponds more to a human chemotherapy, than a single high-dose model. The respective model consists of four intraperitoneal injections of paclitaxel (2 mg/kg) every other day, resulting in a cumulative dose of 8 mg/kg to induce paclitaxel-mediated neuropathic pain (Fig. 1A) [26].

As a first approach, the bioavailability and brain permeability of SAFit2 was monitored in a pharmacokinetic study. Here, we tested two SAFit2 doses and two formulations, measuring SAFit2 concentrations in plasma and brain after treatment (Additional file 1: Fig. S2). SAFit2 was either formulated in PBS supplemented with 5% PEG400, 5% Tween and 0.7% ethanol or as slow-release formulation in vesicular phospholipid gel (VPG, Additional file 1: Fig. S2). We observed that the slow-release VPG formulation led to a strong but transient brain permeability of SAFit2 that did not differ between the two doses or vehicles at later time points.

To analyze the effect of SAFit2 on paclitaxel-induced peripheral neuropathy, we aimed to achieve moderate to high SAFit2 plasma concentrations. However, a single injection of the slow-release formulation was not sufficient to achieve sustained moderate to high plasma concentrations and a single dose of 40 mg/kg SAFit2 led to exceedingly high plasma concentrations of SAFit2 at early time points (Additional file 1: Fig. S2). Based on these results and previous studies in which SAFit2 had been administered in vivo, we decided to treat the animals intraperitoneally with either 10 mg/kg SAFit2 or vehicle two times daily from day five to ten to investigate the therapeutic effect of SAFit2 on paclitaxel-treated mice [14, 24, 27]. After 12 days, the animals were killed and the lumbar DRGs (L1–L6) as well as the respective innervated segments of the spinal cord were isolated and collected separately from each individual animal for further biochemical analysis (Fig. 1A).

To investigate whether SAFit2 has an effect on paclitaxel-mediated neuropathic pain, the pain behavior of mice was assessed before the initiation of the model and during the treatment phase until day twelve by recording the mechanical paw withdrawal threshold (Fig. 1B). Both groups (vehicle and SAFit2) developed a mechanical hypersensitivity over time due to the paclitaxel treatment. However, the SAFit2-treated animals recovered from the mechanical hypersensitivity as their paw withdrawal thresholds significantly increased after the first dose of SAFit2 (Fig. 1B). In addition, the amelioration of pain sensation was sustained after the last treatment of SAFit2 until day twelve (Fig. 1B). In summary, SAFit2 significantly reduced the paclitaxel-induced mechanical hypersensitivity in vivo.

### SAFit2 reduces neuronal stress marker expression

**Fig. 2:**
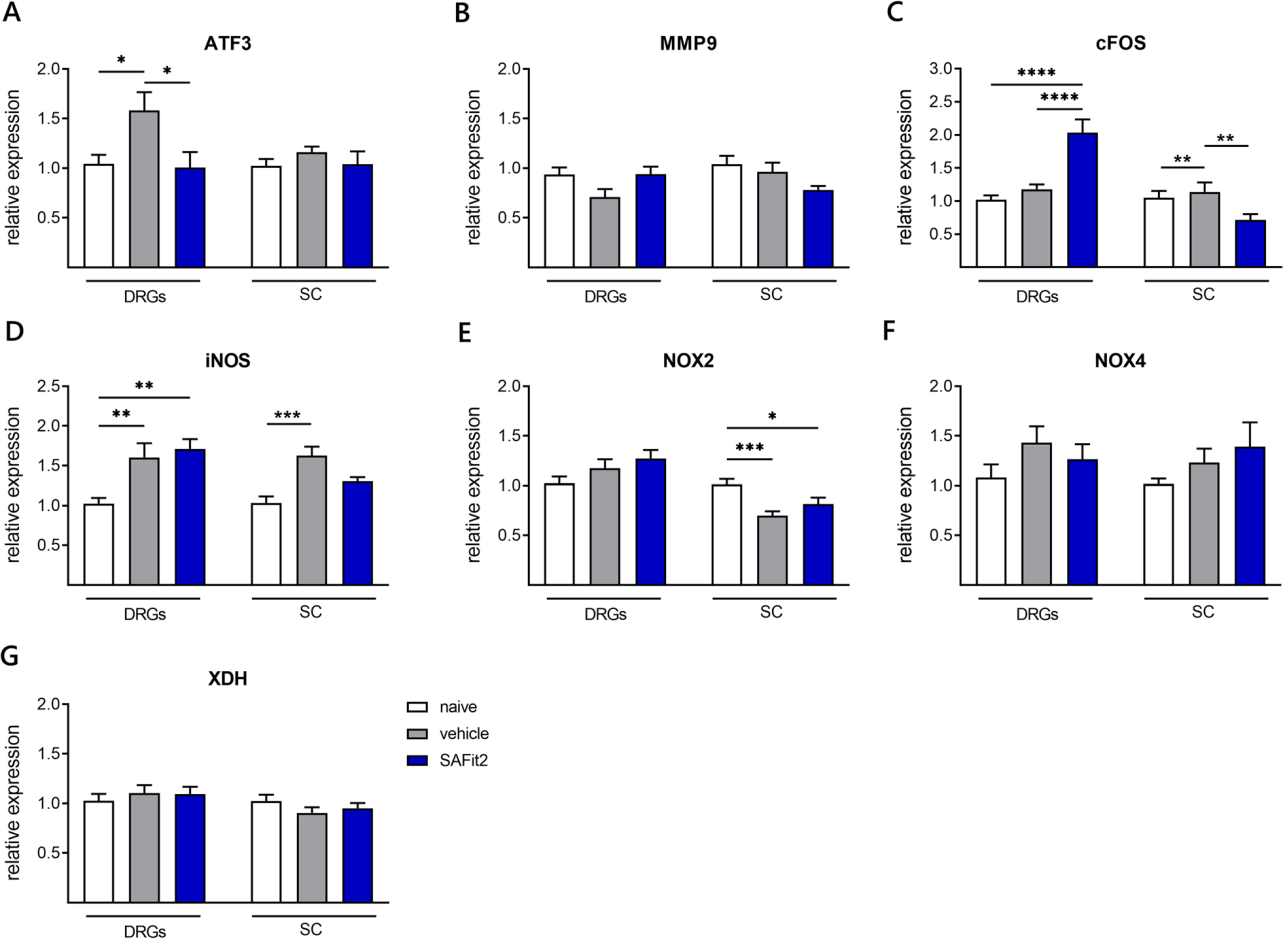
Neuronal stress and ROS marker expression in DRGs and spinal cord. After 12 days, the expression of neuronal stress markers (**A**–**C**) and ROS markers (**D**–**G**) was assessed via qPCR. The expression of vehicle and SAFit2-treated animals were normalized to the expression of naïve animals. The data represent the mean ± SEM from 3–4 mice per group, measured in technical triplicates, respectively. * *p* < 0.05, ** *p* < 0.01, *** *p* < 0.001 one-way ANOVA with Tukey´s multiple comparison test per tissue. *DRGs* dorsal root ganglia, *SC* spinal cord, *SAFit2* selective antagonist of FKBP51 by induced fit 2, *ROS* reactive oxygen species, *ATF3* activating transcription factor 3, *MMP9* matrix metallopeptidase 9, *XDH* xanthine dehydrogenase, *NOX2/4* NADPH oxidase 2/4, *iNOS* inducible nitric oxide synthase

As we detected a significant and sustained pain relief through the treatment with SAFit2, we analyzed the expression of neuronal stress markers (Fig. 2A–C) and reactive oxygen species (ROS) (Fig. 2D–G) markers in DRG and spinal cord samples in order to understand how SAFit2 affected underlying mechanisms. In addition, we compared the gene expression of both paclitaxel-treated groups with the basal expression of naïve mice, termed control group in the following.

For the neuronal stress marker activating transcription factor 3 (ATF3), we detected a significantly reduced expression in the DRGs of SAFit2-treated animals compared to vehicle-treated animals (Fig. 2A). In addition, the downregulation of ATF3 was confirmed via an immunohistochemistry staining in L4 and L5 DRGs of SAFit2-treated animals (Additional file 1: Fig. S3). Furthermore, we detected no significant changes in the expression of matrix metallopeptidase 9 (MMP9) (Fig. 2B). In contrast, we detected significantly diverging changes in the expression of the neuronal activity marker cFOS for both tissues (Fig. 2C). However, we observed on a protein level a significant downregulation of cFOS for both tissues after SAFit2 treatment, which we suggest to occur due to posttranscriptional modifications (Additional file 1: Fig. S4). In general, cFOS is known to be biphasically expressed and to contribute to the resolution of neuronal damage as its expression is enhanced in the formation of neuronal and synaptic plasticity [28], however its early upregulation was associated with nociceptive activity. In summary, we detected an enhanced expression of cFOS on RNA level in the DRGs and a reduced expression of cFOS on protein level in both tissues after SAFit2 treatment. Furthermore, we observed a reduced expression of ATF3 as neuronal stress marker on RNA and protein level in the DRGs after SAFit2 treatment, which indicates a reduction of neuronal stress after paclitaxel treatment.

Furthermore, we analyzed the expression of ROS markers in lumbar DRGs and spinal cord as the cytostatic paclitaxel is known to induce oxidative stress in sensory neurons [29]. However, we only detected a significant increase in the inducible nitric oxide synthase (Fig. 2D). In line with that, we observed no significant differences in the expression of NADPH oxidase two in the DRGs (Fig. 2E) as well as NADPH oxidase four (Fig. 2F) and the xanthine dehydrogenase (Fig. 2G) in both tissues. From this data, we suggested that the time point of investigation, which was day 12 and already 6 days after the last paclitaxel treatment, may be too late to measure alterations in ROS responding genes and that their expression changes may be more transient after paclitaxel treatment.

### SAFit2 reduces pain-mediating chemokine levels and increases anti-inflammatory cytokine levels in DRGs and spinal cord after paclitaxel treatment

**Fig. 3:**
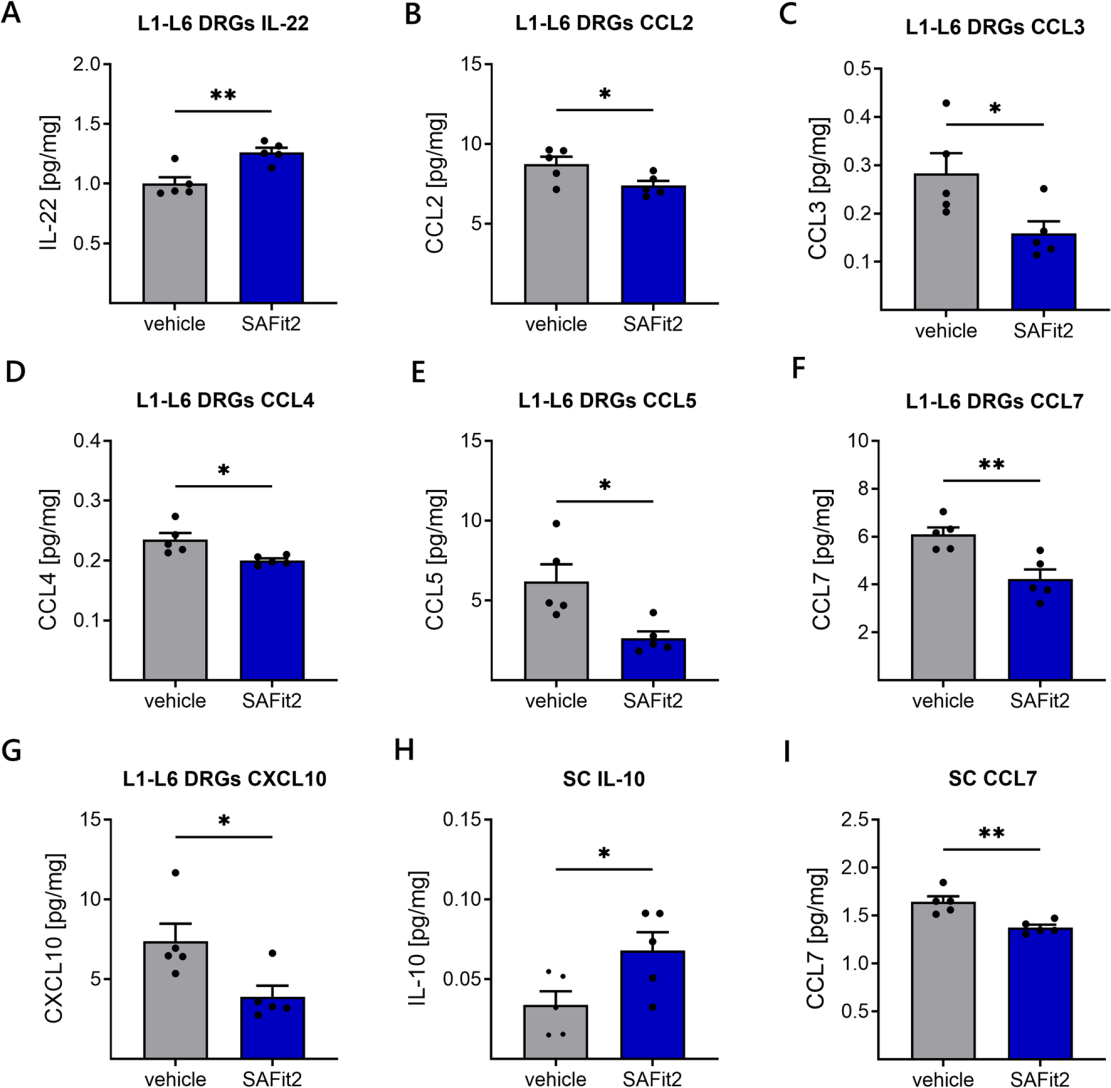
Increase of anti-inflammatory cytokines and decrease of pain-mediating chemokines in neuronal tissue of SAFit2-treated animals. After 12 days, DRGs and spinal cord samples were homogenized and analyzed using a multiplex immunoassay including a panel of 26 cytokines and chemokines. This figure displays a section of significantly altered cytokines and chemokines in the DRGs (**A**–**G**) and spinal cord (**H**, **I**) after vehicle and SAFit2 treatment. The data represent the mean ± SEM from 5 mice per group. The raw data were related to the total protein amount of the sample. * *p* < 0.05, ** *p* < 0.01, *** *p* < 0.001 Student’s *t*-test with Welch’s correction. *SAFit2* selective antagonist of FKBP51 by induced fit 2, *DRGs* dorsal root ganglia, *SC* spinal cord, *IL* interleukin, *CCL* C–C motif ligand, *CXCL 10* C-X-C ligand 10

Since we did not observe strong differences in the gene expression of neuronal stress and ROS markers except for ATF3 and cFOS after SAFit2 treatment, we next measured the concentrations of inflammatory and proalgesic mediators in the DRGs and the respective segments of the spinal cord. Therefore, we performed a multiplex immunoassay assay, comprising a panel of 26 mediators, to determine differences in the neuroinflammation between vehicle and SAFit2-treated animals in a multiple low-dose paclitaxel model (Fig. 3, Additional file 1: Figs. S5, S6, S7).

Analyzing these mediators, we observed a significant upregulation of the anti-inflammatory interleukin 22 in the DRGs (Fig. 3A) as well as a significant downregulation of several pain-mediating chemokines such as CCL 2, CCL3, CCL4, CCL5, CCL7 and CXCL10 after SAFit2 treatment (Fig. 3B–G). Moreover, we detected the same tendencies in the spinal cord, since we observed a significant upregulation of the anti-inflammatory cytokine interleukin 10 (Fig. 3H) and the significant downregulation of CCL7 (Fig. 3I). Overall, SAFit2 seems to decrease the levels of pain-mediating chemokines and to increase anti-inflammatory mediators in both neural tissues after paclitaxel treatment.

### SAFit2 treatment decreases activation of astrocytes and microglia in the spinal cord of paclitaxel-treated mice

**Fig. 4:**
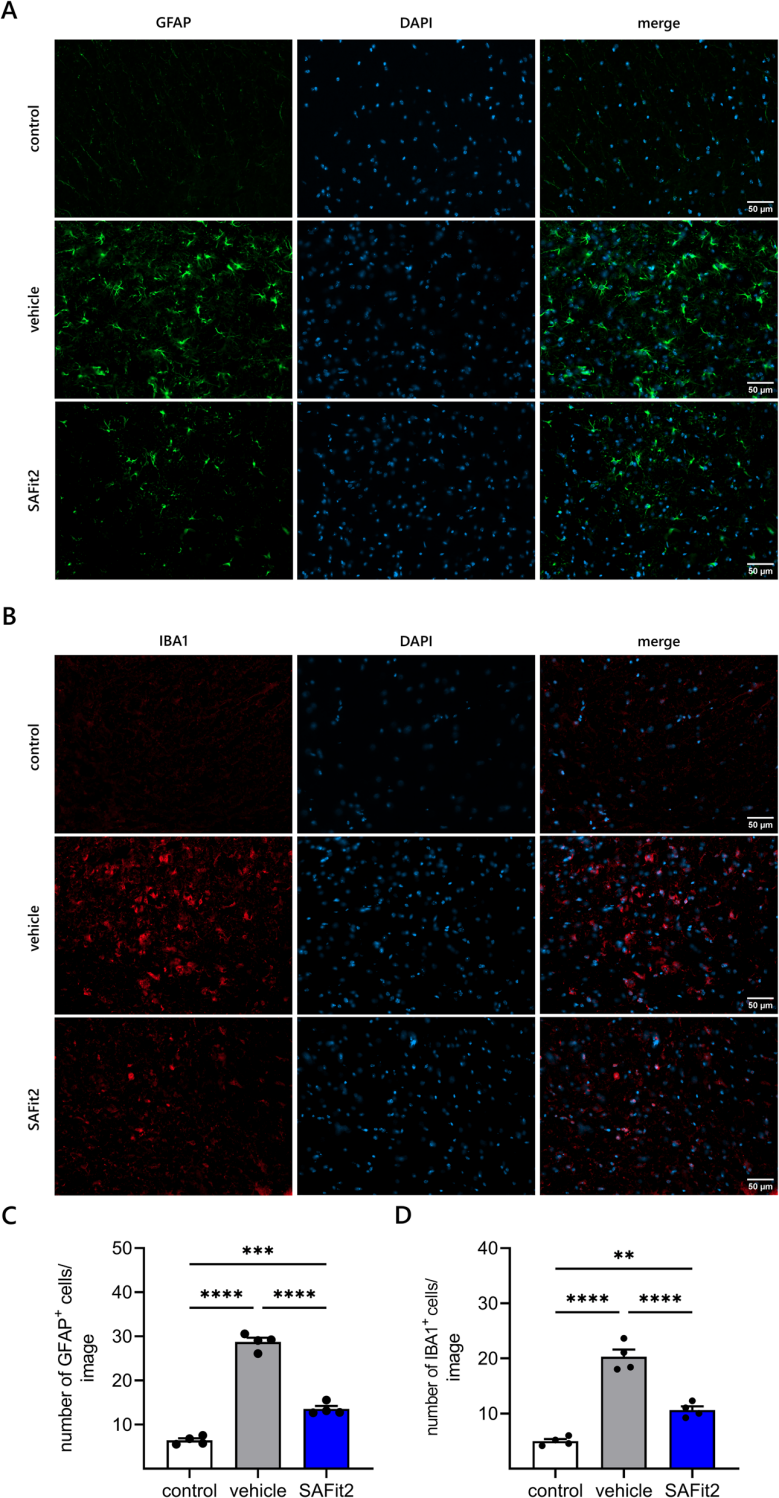
Reduced activation of astrocytes and microglia in the spinal cord of SAFit2-treated animals. Immunohistochemistry staining of astrocyte activation marker GFAP (**A**) and microglia marker IBA1 (**B**) at 20X magnification (scale bar: 50 µm) of spinal cord (dorsal horn) slices. Samples of naïve animals were labeled as control. **C** Quantification of GFAP-positive cells. **D** Quantification of IBA1-positive cells. **C**, **D** Positive cells were counted in 15–20 images per animal and averaged. Data represent the mean ± SEM from 15–20 images of four mice per group. ** *p* < 0.01, *** *p* < 0.001, **** *p* < 0.0001 one-way ANOVA with Tukey’s multiple comparison test. *SAFit2* selective antagonist of FKBP51 by induced fit 2, *GFAP* glial fibrillary acidic protein, *IBA1* ionized calcium-binding adaptor molecule 1

Based on the strong effect of SAFit2 on pain-mediating chemokines in the lumbar DRGs and spinal cord, we investigated the effect of SAFit2 regarding spinal gliosis, since activated astrocytes and microglia are the predominant source of cytokines and chemokines in the spinal cord [30, 31]. To assess this, we performed immunohistochemical stainings of dorsal horn samples to assess the activation of astrocytes and the number of microglia (Fig. 4).

First, we analyzed the activation of astrocytes with the astrocyte activation marker glial fibrillary acidic protein (GFAP) in dorsal horn cryo-slices (Fig. 4A, C). In these stainings, we could observe a high number of GFAP-positive cells in the vehicle-treated paclitaxel group, indicating a strong activation of astrocytes after paclitaxel treatment (Fig. 4A). In contrast, the number of GFAP-positive cells was lower in the SAFit2-treated paclitaxel group. The control group, which consists of naïve animals, showed no or only a little GFAP-positive cells in comparison. In summary, the staining quantification confirmed these observations and revealed a significant reduction about 50% of astrocyte activation after SAFit2 treatment (Fig. 4D).

Next, we assessed the expression of the microglia marker ionized calcium-binding adaptor molecule 1 (IBA1) in the dorsal horn of the respective groups (Fig. 4B, D). The staining of the vehicle-treated paclitaxel group showed a strong IBA1 expression (Fig. 4B). However, the staining of the SAFit2-treated paclitaxel group showed less IBA1-positive cells. Stainings of the control group showed only weak signals, as expected. Interestingly, the quantification of IBA1-positive cells also revealed a significant reduction of approximately 50% after SAFit2 treatment. In conclusion, we observed that a SAFit2 treatment significantly reduced the number of activated astrocytes and microglia in the spinal cord, indicating that SAFit2 counteracts the paclitaxel-mediated gliosis.

### SAFit2 treatment leads to increased free fatty acid levels in neuronal tissue

Lipid mediators are important signaling mediators in the context of neuropathic pain as they can be released as paracrine signaling molecules to interact with neurons and glial cells as well as to modulate and resolve neuroinflammation [32]. Based on that, we hypothesized that SAFit2 might have an influence on lipid synthesis or release. For assessing this hypothesis, we first performed a high-resolution mass spectrometry lipidomic screen (LC–HRMS) to investigate lipid levels in nervous tissue (lumbar DRG and spinal cord samples) on a broad scale comparing vehicle and SAFit2-treated paclitaxel animals.

**Fig. 5:**
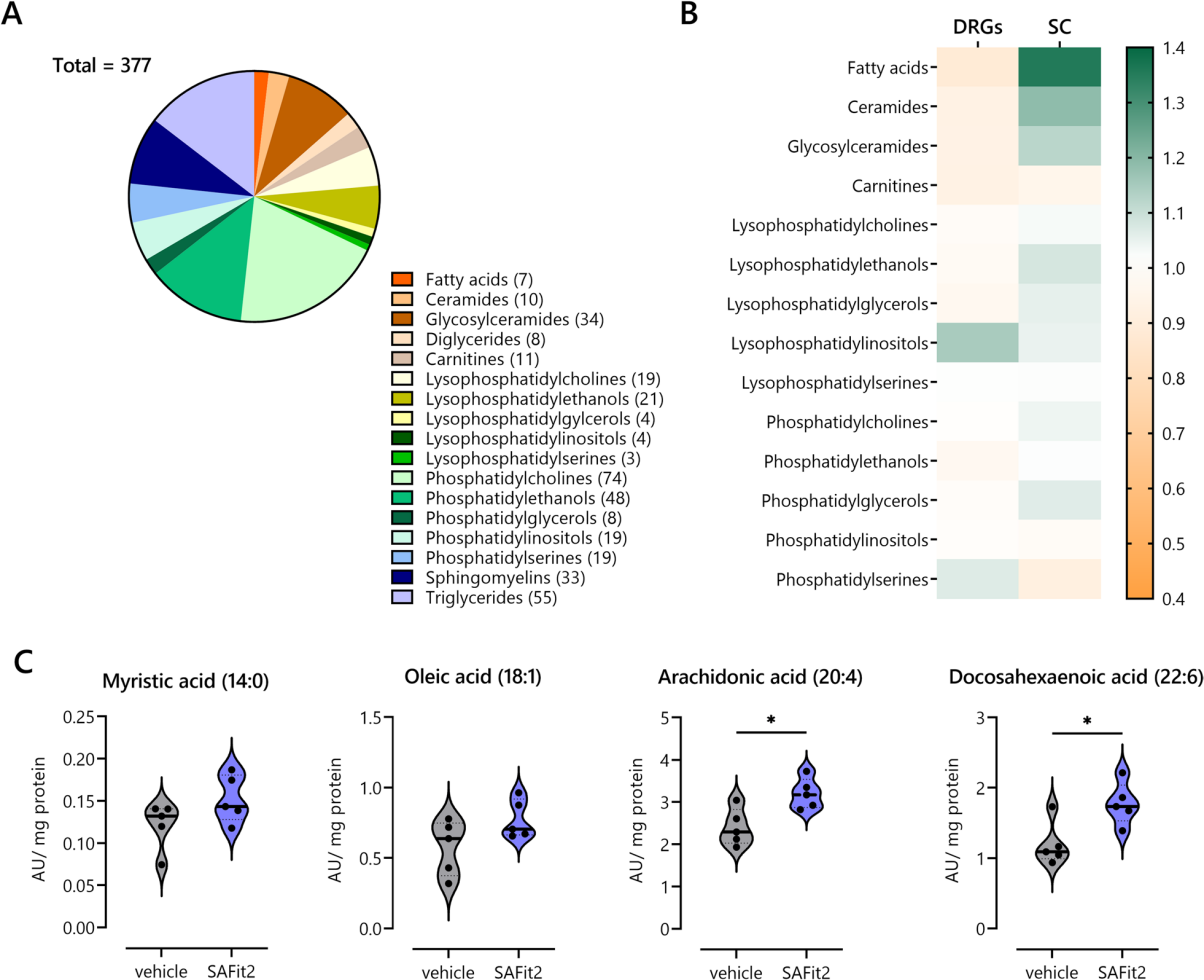
LC–HRMS lipid analysis of DRGs and spinal cord from vehicle and SAFit2-treated paclitaxel mice. **A** Distribution of measured lipid classes of both tissues in LC–HRMS analysis. **B** Relative frequencies of lipid classes in lumbar DRGs and spinal cord from SAFit2-treated animals, which were normalized to those of vehicle-treated animals. **C** Violin plots comparing fatty acid levels of spinal cord samples from vehicle and SAFit2-treated animals. The data represent the mean ± SEM from five mice per group. * *p* < 0.05 Student’s *t*-test with Welch’s correction. *DRGs* dorsal root ganglia, *SAFit2* selective antagonist of FKBP51 by induced fit 2, *LC–HRMS* liquid chromatography–high-resolution mass spectrometry

In this lipid screen, we detected 377 individual lipids that can be further subdivided into the following seventeen lipid classes: fatty acids, ceramides, glycosylceramides, diglycerides, carnitines, lysophosphatidylcholines, -ethanols, -glycerols, -inositols, -serines, phosphatidylcholines, -ethanols, -glycerols, -inositols, -serines, sphingomyelins and triglycerides (Fig. 5A). For further analysis, the values of SAFit2-treated animals were normalized to the values of vehicle-treated animals. Afterwards, the normalized values were compared class wise for DRG and spinal cord samples. Focusing on lipid classes that include potential lipid mediators, we observed a slight increase of lysophosphatidylinositols in the DRGs and ceramides as well as glycosylceramides in the spinal cord after SAFit2 treatment (Fig. 5B). However, we detected a strong increase of fatty acids in the spinal cord through the treatment of SAFit2 (Fig. 5B).

Since the class of fatty acids was strongly elevated after SAFit2 treatment and has a particular relevance in the context of neuropathic pain, we further determined differences between the groups for individual fatty acids. Thereby, we detected increased levels of all measurable fatty acids in the spinal cord. The most drastically changed fatty acids were arachidonic acid (20:4) and docosahexaenoic acid (22:6). We also observed a slight upregulation of myristic acid (14:0) and oleic acid (18:1) (Fig. 5C). In summary, we detected indeed an elevation of free fatty acids and especially of polyunsaturated fatty acids (PUFAs) after SAFit2 treatment, which can be rapidly metabolized into pain-relevant signaling mediators.

**Fig. 6:**
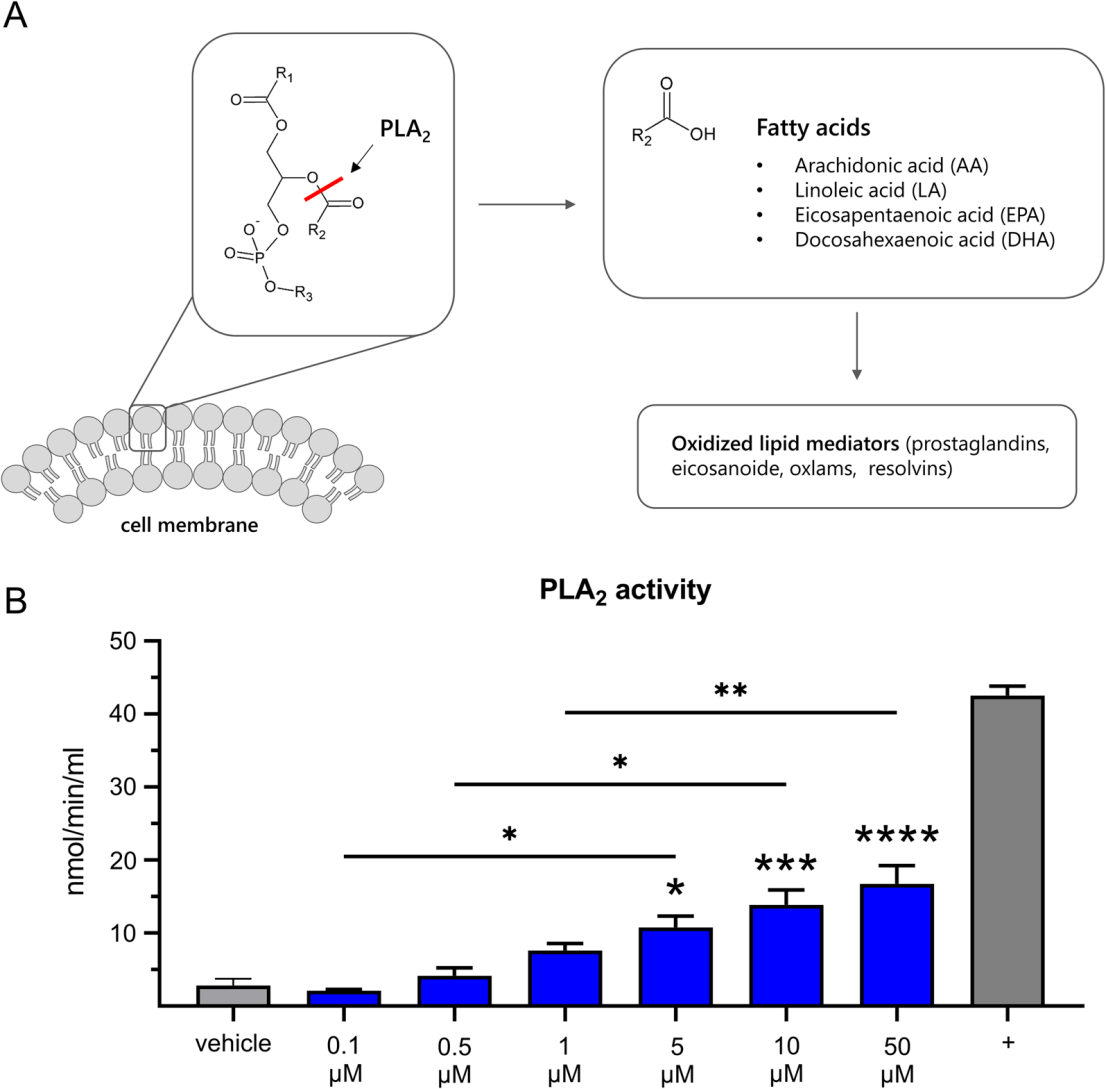
Influence of SAFit2 on phospholipase 2 activity in primary sensory neuron lysates. **A** Schematic illustration of the phospholipase 2 function and its products. Phospholipase 2 hydrolyzes the second ester bond of phospholipids that constitute the lipid bilayer of cell membranes. Thereby, polyunsaturated fatty acids were released, which were rapidly metabolized into oxylipins. **B** The influence of SAFit2 (0.1–50 µM) on the phospholipase 2 activity was assessed in lysed primary sensory neurons. Further, the basal phospholipase activity of the lysates was determined, the influence of the solvent DMSO (vehicle) verified and bee venom used as a positive control. Data represent the mean ± SEM. * *p* < 0.05, ** *p* < 0.01, *** *p* < 0.001, **** *p* < 0.0001 one-way ANOVA with Tukey’s post-multiple comparison test. *PLA*_*2*_ phospholipase 2, *SAFit2* selective antagonist of FKBP51 by induced fit 2

PUFAs can be absorbed from nutrition, but they can also be released from the lipid bilayer of cell membranes by the hydrolysis of an ester bond (Fig. 6A). In particular, the phospholipase 2 can hydrolase the ester bond from the second carbon of a phospholipid, whereby a fatty acid is released. This free fatty acid can consist of different chain length, however, arachidonic acid, linoleic acid, eicosapentaenoic acid and docosahexaenoic acid are the most abundant ones. Nevertheless, these PUFAs are usually rapidly metabolized into both pro- and anti-inflammatory mediators that can regulate and influence the transmission of neuropathic pain [16]. To analyze whether SAFit2 can enhance the release of PUFAs, we investigated the effect of SAFit2 on the phospholipase 2 activity in vitro. Interestingly, SAFit2 contributes to an enhanced activity of the phospholipase 2 isoforms cPLA_2_, iPLA_2_ and sPLA_2_ in primary sensory neuron lysates as we observed a concentration dependent increase of hydrolyzed substrate due to increasing SAFit2 concentrations (Fig. 6B). In summary, we observed that SAFit2 increases PUFA levels in vivo and contributes to the phospholipase 2 mediated release of PUFAs from neuronal membranes.

### SAFit2 enhances pro-resolving mediators after paclitaxel treatment

**Fig. 7:**
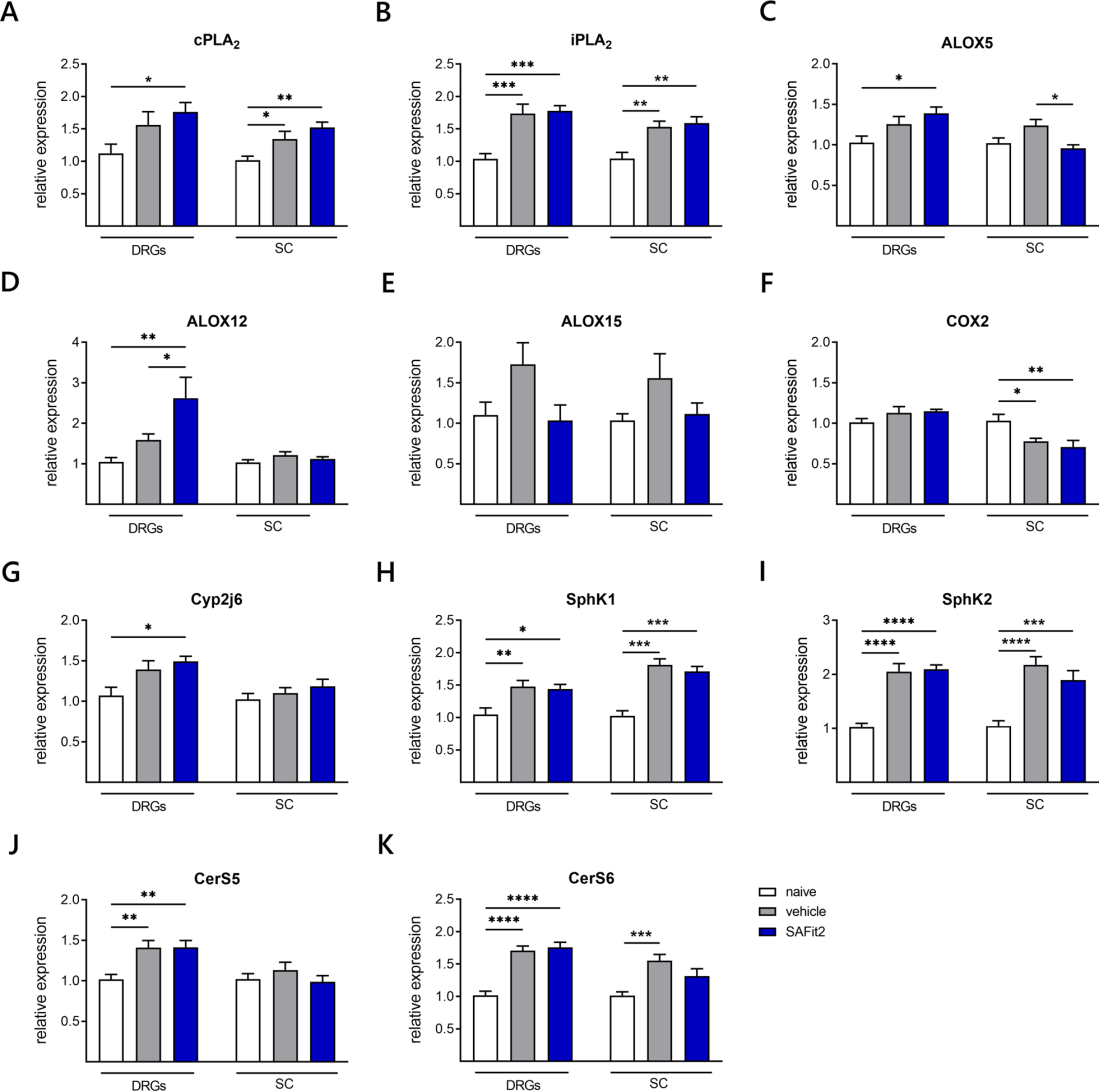
Gene expression of lipid synthesizing and metabolizing enzymes in neuronal tissue of SAFit2-treated paclitaxel mice. After twelve days, the expression of phospholipases (**A**, **B**), lipoxygenases (**C**–**F**), epoxygenase (**G**), sphingosine kinases (**H**, **I**) and ceramide synthases (**J**, **K**) was assessed via qPCR. The expression levels of vehicle and SAFit2-treated animals were normalized to the expression levels of naïve animals. The data represent the mean ± SEM from 3–4 mice per group, measured in technical triplicates, respectively. * *p* < 0.05, ** *p* < 0.01, *** *p* < 0.001 one-way ANOVA with Tukey´s post hoc test was conducted per tissue. *DRGs* dorsal root ganglia, *SC* spinal cord, *SAFit2* selective antagonist of FKBP51 by induced fit 2, *cPLA*_*2*_ cytosolic phospholipase A2, *iPLA*_*2*_ calcium-independent phospholipase A2, *ALOX* arachidonate lipoxygenase, *CYP2J6* cytochrome P450 family 2 subfamily j polypeptide 6c, *COX2* cyclooxygenase 2, *SphK1/2* sphingosine kinase 1/ 2, *CerS* ceramide synthase

As a next step, we investigated the expression of phospholipases and fatty acid metabolizing enzymes as we have observed an increase of free fatty acids after SAFit2 treatment (Fig. 7A–G). Furthermore, we compared the expression of both vehicle and SAFit2-treated paclitaxel groups with the expression of naïve animals.

Consistent with our previous results, we observed an increased expression of the phospholipases cPLA_2_ and iPLA_2_ in SAFti2-treated paclitaxel animals compared to the expression of control animals (Fig. 7A, B). In addition, we measured a significant upregulation of the lipoxygenase 5 (ALOX5) in the DRGs of SAFit2-treated paclitaxel animals (Fig. 7C). In contrast, the expression of this enzyme was significantly lower in the spinal cord of SAFit2-treated animals than in the vehicle-treated paclitaxel animals, yet the expression of both paclitaxel groups was not significantly altered from the control group. Nevertheless, the lipoxygenase 12 (ALOX12) was significantly increased in the DRGs of the SAFit2-treated group compared with both vehicle and control group (Fig. 7D). The expression of the lipoxygenase 15 (ALOX15) was not significantly altered in both tissues (Fig. 7E) and the expression of the cyclooxygenase 2 (COX2) was significantly reduced in the spinal cord for both paclitaxel groups compared to the vehicle group (Fig. 7F). However, we observed a significant increase of the cytochrome-P_450_-epoxygenase CYP2J6 in the DRGs after SAFit2 treatment (Fig. 7G). The expression of the cytochrome-P_450_-epoxygenase CYP3A11 was lower than the detection limit in both tissues. In summary, we could observe significant differences in the expression of lipoxygenases and CYP2J6 comparing vehicle and SAFit2-treated paclitaxel animals.

Furthermore, we analyzed the expression of sphingosine kinases and ceramide synthases to detect any SAFit2-mediated alterations (Fig. 7F–K). However, we detected no significant changes in expression of vehicle and SAFit2-treated paclitaxel animals for the sphingosine kinases one and two, though both groups were significantly increased compared to the control group (Fig. 7H, I). In addition, we observed no significant differences in the expression of ceramide synthase five and six for vehicle and SAFit2-treated paclitaxel mice (Fig. 7J, K). In summary, we detected a significant increase in the expression of fatty acid oxidizing and metabolizing enzymes in the DRGs and spinal cord of SAFit2-treated animals.

**Fig. 8:**
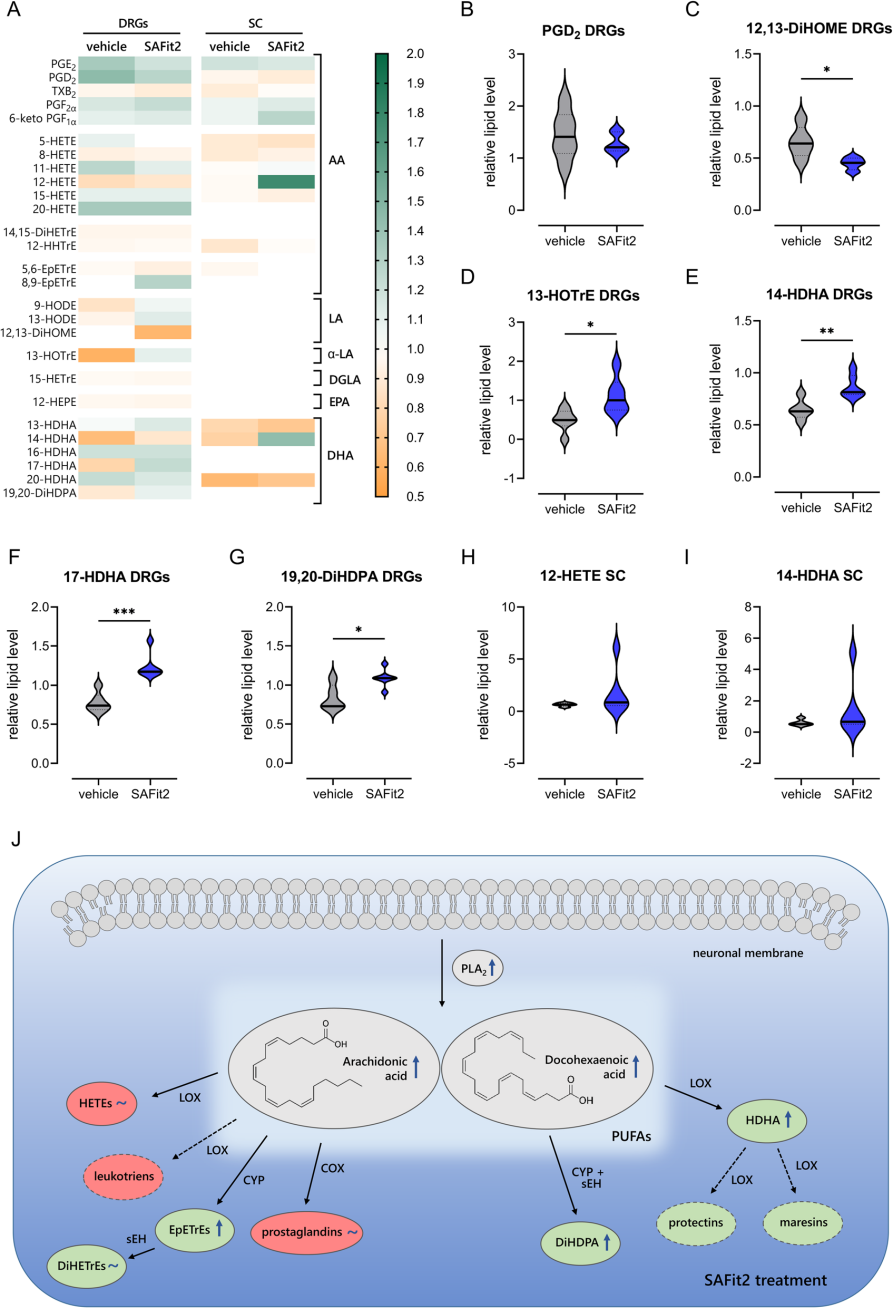
Alteration of oxylipins in DRGs and spinal cord of vehicle and SAFit2-treated paclitaxel mice. **A** Relative amount of oxylipins was measured in the DRGs and spinal cord of both groups and clustered in their precursor fatty acids. The raw data were normalized to the protein amount of the respective sample. **B**–**I** Violin plots display the relative lipid levels of those lipids, which were altered after SAFit2 treatment. The measured lipid levels of paclitaxel-treated animals were normalized to lipid levels of naïve animals. The analysis was conducted with six animals per group. * *p* < 0.05, ** *p* < 0.01, *** *p* < 0.001 student’s *t*-test with Welch´s correction. **J** Schematic summary of the effect of SAFit2 on lipid levels. SAFit2 seems to enhance the PLA_2_ mediated fatty acid release from phospholipids. In particular, we detected an elevation of arachidonic acid and docosahexaenoic acid after SAFit2 treatment as well as an increase of their pro-resolving metabolites. Pro-inflammatory oxylipins were marked in red, anti-inflammatory/ pro-resolving in green, the alteration after SAFit2 treatment is indicated by either a blue arrow for an increase or a blue tilde for no changes. Oxylipins indicated with dashed lines were below the detection limit. *DRG* dorsal root ganglia, *SC* spinal cord, *SAFit2*: selective antagonist of FKBP51 by induced fit 2, *AA* arachidonic acid, *LA* linoleic acid, *α-LA* alpha-linoleic acid, *DGLA* dihomo-γ-linolenic acid, *EPA* eicosapentaenoic acid, *DHA* docosahexaenoic acid, *PGE*_*2*_ prostaglandin E2, *PGD*_*2*_ prostaglandin D2, *TXB*_*2*_ thromboxane 2, *PGF2α* prostaglandin F2 alpha, *6-keto PGF*_*1α*_ 6-keto prostaglandin F1 alpha, *HETE* hydroxyeicosatetraenoic acid, *DiHETrE* dihydroxyeicosatrienoic acid, *HHTrE* hydroxyheptadecatrienoic acid, *EpETrE* hydroxyepoxyeicosatrienoic acid, *HODE* hydroxyoctadecadienoic acid, *DiHOME* dihydroxyoctadecenoic acid, *HOTrE* hydroxyoctadecatrienoic acid, *HETrE* hydroxyeicosatrienoic acid. *HEPE* hydroxyeicosapentaenoic acid, *HDHA* hydroxydocosahexaenoic acid, *DiHDPA*: dihydroxydocosapentaenoic acid

Since we observed an increased PLA_2_ activity, elevated free fatty acid levels and an increased expression of fatty acid metabolizing enzymes after SAFit2 treatment, we further investigated the influence of SAFit2 on oxylipin levels. Oxylipins are mediators that are generated by the oxidation of free fatty acids and have been shown to play a crucial role as signaling molecules in the pathophysiological context of neuropathic pain [16, 33, 34]. To assess oxylipin levels of vehicle and SAFit2-treated paclitaxel mice as well as naïve mice, we performed a targeted LC–MS/MS analysis with DRG and spinal cord samples of the respective groups in which we were able to detect 27 oxylipins. Afterwards, the oxylipin levels of paclitaxel-treated animals were normalized to those of the control group and clustered in their precursor fatty acids (Fig. 8).

For the arachidonic acid metabolites, we detected a slight decrease of pro-inflammatory prostaglandins in the DRGs after SAFit2 treatment (Fig. 8A), which is shown representatively for prostaglandin D2 (Fig. 8B). Furthermore, we detected an increase of the anti-inflammatory and pro-resolving mediator 8,9-EpETrE in the DRGs. For the linoleic acid and alpha linoleic acid metabolites, we detected a significant decrease of the pro-inflammatory 12,13-DiHOME (Fig. 8C) and a significant increase of the anti-inflammatory 13-HOTrE after SAFit2 treatment (Fig. 8D). Consistently, we detected a significant increase of three docosahexaenoic acid metabolites 14-HDHA, 17-HDHA, 19.20-DiHDPA (Fig. 8E–G), which have also anti-inflammatory and pro-resolving properties. For the spinal cord samples, we could observe an increase of 12-HETE after SAFit2 treatment (Fig. 8A). However, 12-HETE was not significantly increased after SAFit2 treatment (Fig. 8H). Moreover, we measured a slight increase of 14-HDHA after SAFit2 treatment (Fig. 8I). In summary, we observed a decrease of pro-inflammatory and an increase of anti-inflammatory and pro-resolving oxylipins in the DRGs and spinal cord after the treatment with SAFit2.

In conclusion, we observed that SAFit2 enhances the PLA_2_ mediated fatty acid release from neuronal membranes in paclitaxel-mediated pain states (Fig. 8J). Thereby, predominantly the PUFAs arachidonic acid and docosahexaenoic acid were released and oxidized into oxylipins, which have previously been shown to influence and regulate neuropathic pain persistence and transmission. Interestingly, we mainly observed an increase of anti-inflammatory and pro-resolving fatty acid metabolites after SAFit2 treatment. Based on these results, we suggest that SAFit2 treatment leads to an increase of pro-resolving lipid mediators, which counteract neuroinflammation and spinal gliosis, resulting in an amelioration of paclitaxel-mediated neuropathic pain.

## Discussion

The need for CIPN therapeutics is increasing nowadays as available treatment strategies have either a low efficacy or severe side effects [35]. Interestingly, our data show that the novel and specific FKBP51 inhibitor SAFit2 can efficiently reduce mechanical hypersensitivity in paclitaxel-treated mice as it counteracts the paclitaxel-induced peripheral sensitization and neuroinflammation.

### SAFit2 reduces paclitaxel-induced peripheral sensitization

Firstly, we observed that SAFit2 has an influence on endogenous lipid levels as it decreases the levels of pro-inflammatory and transient receptor potential (TRP) channel sensitizing lipids mediators. More specifically, we detected that SAFit2 significantly reduces the levels of 12,13-DiHOME, which is a pro-inflammatory diol lipid that is metabolized from linoleic acid-derived epoxylipids. Furthermore, 12,13-DiHOME is known to mediate thermal hyperalgesia in mice as it sensitizes the pain-mediating TRPV1 channel and thereby increases the excitability of sensory neurons under inflammatory conditions [36]. In line with that, eicosanoids like the prostaglandin E_2_ are also known to sensitize TRP channels and thereby enhance the excitability of sensory neurons [37, 38]. However, we detected a slight decrease of the prostaglandins E_2_ and D_2_ after SAFit2 treatment. A possible explanation for the decreased prostaglandin levels might be a reduced expression of COX2 after SAFit2 treatment, since prostaglandins are predominantly synthesized by the COX2 enzyme [39, 40]. Based on this observation, we assume that a SAFit2 treatment mediates a more anti-inflammatory lipid profile, by shifting oxylipin synthesis towards the LOX and CYP enzymes and not towards COX2, which might reveal one mechanism how SAFit2 is capable to counteract the enhanced excitability of sensory neurons after paclitaxel treatment.

### SAFit2 induces the resolution of paclitaxel-induced neuroinflammation

Peripheral sensitization can also be initiated and regulated by pain-mediating chemokines and pro-inflammatory mediators, that are released from activated glial cells [41]. In addition, it has been shown that paclitaxel-activated glial cells such as microglia and astrocytes can become neurotoxic [3]. Nevertheless, we observed that SAFit2 is capable to reduce gliosis after paclitaxel treatment, which leads to a reduction of pro-inflammatory and pain-mediating cytokines and chemokines in the spinal cord. In line with that, we detected a strong decrease of chemokines such as CCL2 and CCL7, which both are agonists of the C–C chemokine receptor type 2 that is expressed by both sensory neurons and glial cells [42]. The decrease of these chemokines has an essential influence on the pain perception of mice as CCL2 was shown to increase the frequency of excitatory post synaptic currents as well as to enhance the currents of AMPA and NMDA receptors, leading to an enhanced excitability of neurons and a sensitized pain state [43, 44]. In addition, both CCL7 and CCL2 have been shown to be direct activators of microglia [45, 46]. Another crucial pain-mediating chemokine, which is strongly downregulated after SAFit2 treatment, is CCL5, which was previously shown to induce hyperalgesia dose-dependently in mice [47–49]. Although some of the detected inflammatory mediators can be secreted from neurons (CCL2 and CXCL10) [32, 50], most of these mediators are released by immune cells, such as astrocytes (CCL2, CCL5, CCL7, CXCL10, IL-10), microglia (CCL5, CXCL10, IL-10) macrophages (CCL3, CCL4, IL-10) and T-cells (CCL4, IL-22) [51–57].

We did not investigate the levels of immune cells in neuronal tissue after SAFit2 treatment. However, the combined cytokine and chemokine data indicate a reduction of pro-inflammatory macrophages and an increase of anti-inflammatory macrophages and T-cells in DRGs after SAFit2 treatment. These data are also in line with our observations showing reduced numbers of activated astrocytes and microglia in the dorsal spinal cord after SAFit2 treatment. Mechanistically, we assume that SAFit2 on the one hand reduces the levels of these mediators as it counterbalances the enhanced NF-κB activation [27] and on the other hand causes a more anti-inflammatory lipid profile. Thereby, SAFit2 is capable to decrease neuroinflammation and prevents gliosis and subsequently neurodegeneration in the spinal cord.

Furthermore, emerging evidence indicates that the resolution of neuroinflammation, which is mediated by pro-resolving mediators, becomes an essential issue in the relief of neuropathic pain [58–60]. In line with that, we detected an increase of anti-inflammatory cytokines like interleukin 10 and 22 after the treatment with SAFit2, indicating that SAFit2 even induces a resolution of neuroinflammation, which contributes to a relief of paclitaxel-mediated neuropathic pain.

### SAFit2 causes a shift towards an anti-inflammatory lipid profile in nervous tissue

Besides anti-inflammatory cytokines, PUFAs, such as DHA, have been shown to comprise anti-inflammatory and pro-resolving properties [61]. Interestingly, we observed an enhanced expression and activity of phospholipase 2 after SAFit2 treatment, which resulted in an increase of free polyunsaturated fatty acids and especially of the omega-3 PUFA DHA. These results further confirm our previously suggested SAFit2-mediated anti-inflammatory lipid profile, as DHA was shown to reduce neuropathic pain in a diabetic rat model as well as in a nerve injury rat and mouse model [62]. Moreover, studies reported that DHA has a protective effect on microglia and astrocytes as it counteracts oxidative stress induced calcium dysregulations and ER stress [63, 64].

Nevertheless, PUFAs such as DHA are known to be rapidly metabolized into oxylipins that play a crucial role in the regulation of neuropathic pain [16, 35]. In particular, the free fatty acid DHA gets metabolized by LOX and CYP enzymes into anti-inflammatory specialized pro-resolving mediators (SPMs) such as resolvins, protectins and maresins [65]. In line with that, we observed beside increased DHA levels also an enhanced expression of the respective LOX and CYP enzymes after SAFit2 treatment. Interestingly, these pro-resolving mediators and especially the class of resolvin mediators was shown to dampen inflammatory pain as they interfere in the mechanism of peripheral sensitization and reduce the excitability of sensory neurons [66]. Furthermore, the SPM neuroprotection D1 was able to reduce spinal gliosis and mechanical hypersensitivity in a neuropathic nerve injury model in vivo [67]. Unfortunately, we were not capable to detect SPMs in the DRG and spinal cord samples. However, we observed an increase of their anti-inflammatory precursors after SAFit2 treatment. More specifically, we detected an increase of hydroxydocosahexaenoic acids (HDHAs) such as 14-HDHA and 17-HDHA after SAFit2 treatment. Based on the elevation of DHA and its metabolites after SAFit2 treatment, we conclude that SAFit2 enhances the levels of pro-resolving DHA-originated lipid mediators, which contribute to an anti-inflammatory lipid profile.

Apart from the DHA-originated mediators, we also detected an increase of the alpha linoleic acid metabolite 13-HOTrE. Although less is known about alpha linoleic acid-derived oxylipins in the literature, 13-HOTrE was associated with anti-inflammatory properties as it was shown to suppress the expression of the inflammatory cytokine IL-1β in human chondrocytes and to reduce glomerulomegaly in obese rats [68]. Moreover, we observed an increase in EpETrEs (e.g., 8,9-EpETrEs) after SAFit2 treatment, which are epoxyeicosatrienoic acids that are generated by the oxidation of arachidonic acid [69]. EpETrEs have also been shown to have anti-inflammatory properties as they inhibit the NF-κB translocation into the nucleus and thereby reduce the expression of, e.g., pro-inflammatory mediators or enzymes, that generate pro-inflammatory mediators, such as COX2 [70]. Based on these results, we assume that SAFit2 induces an anti-inflammatory lipid profile after paclitaxel treatment that reduces peripheral sensitization and consequently reduces paclitaxel-mediated mechanical hypersensitivity.

### Limitations and conclusion

A possible limitation of the study could be the lack of female mice. However, it has been previously shown that the involvement of FKBP51 in neuropathic pain is sex independent and its deficiency leads to an equal pain relief in both sexes [11]. A further limitation could also be the investigation of SAFit2-mediated effects on exclusively the peripheral nervous system and spinal cord regions as these compartments mediate and regulate the development and maintenance of neuropathic pain. Nevertheless, SAFit2 is also capable of affecting FKBP51 levels in the central nervous system as it passes the blood–brain barrier. However, the inhibition of FKBP51 in the brain displays not a disadvantage but rather seems to be beneficial as previous studies have shown that the inhibition of FKBP51 in the brain helps to prevent psychological disorders as well as stress-related and endocrinologic-mediated diseases such as depression, type two diabetes and obesity [71].

Indeed, SAFit2 has already been tested in several neuropathic models and showed promising effects in reducing mechanical and thermal hypersensitivity in vivo. The models themselves differ profoundly concerning the degree of neuroinflammation to pain hypersensitivity. While the nerve injury models SNI and CCI (spared nerve injury, chronic constriction injury) are usually connected with a strong spinal gliosis and related neuroinflammation, the chemotherapy-induced neuropathy models usually show a more neurotoxic and less inflammatory effect, although this depends on the cytostatic drug that is used [32, 72–74].

In line with these reports, we observed elevated levels of cytokines (especially in the dorsal spinal cord) and chemokines (especially in DRGs) in the SNI model, many of which were significantly reduced after SAFit2 treatment [27]. Similar results were observed in animals deficient of FKBP51 [10]. In the current manuscript, we do not see this strong and general increase in (neuro)inflammatory mediators, especially not in pro-inflammatory interleukins, which is in line with previous results [11]. In this model, we observe that SAFit2 rather causes a decrease of pro-inflammatory chemokines in DRGs and an increase of anti-inflammatory mediators (IL-10, IL-22). Moreover, we show, that SAFit2 treatment also causes a shift towards a pro-resolving lipidomic profile within DRGs and the dorsal spinal cord.

In conclusion, we show that the novel and specific FKBP51 inhibitor SAFit2 reduces gliosis in the spinal cord, which leads to a reduction of pain-mediating chemokines. Furthermore, we observed an increase of anti-inflammatory and pro-resolving cytokines and lipid mediators due to a SAFit2 treatment, which contribute to a resolution of neuroinflammation and peripheral sensitization after paclitaxel treatment. In summary, these effects can explain the SAFit2-mediated pain relief after paclitaxel treatment and highlight the role of SAFit2 as potential novel analgesic drug candidate.

## Supplementary Information

**Additional file 1: Figure S1.** Enhanced FKBP51 expression in spinal cord and L4-L5 DRG slices of paclitaxel-treated animals. **Figure S2.** Pharmacokinetic study of SAFit2 comparing two formulations. **Figure S3.** Reduced ATF3 expression in L4 and L5 DRGs of SAFit2-treated animals. **Figure S4.** Reduced cFOS expression in spinal cord and L4-L5 DRG slices of SAFit2-treated animals. **Figure S5.** Cytokines and chemokines measured in the DRGs of paclitaxel-treated mice. **Figure S6.** Cytokines measured in the spinal cord of paclitaxel-treated mice. **Figure S7.** Chemokines measured in the spinal cord of paclitaxel-treated mice.

## Data Availability

All data generated or analyzed during this study are included in this published article and its additional information file.

## Abbreviations

AA: Arachidonic acid
CCL: C-C motif ligand
CIPN: Chemotherapy-induced peripheral neuropathy
CXCL: C-X-C motif ligand
DGLA: Dihomo-γ-linolenic acid
DHA: Docosahexaenoic acid
DiHDPA: Dihydroxydocosapentaenoic acid
DiHETrE: Dihydroxyeicosatrienoic acid
DiHOME: Dihydroxyoctadecenoic acid
DRGs: Dorsal root ganglia
EPA: Eicosapentaenoic acid
EpETrE: Epoxyeicosatrienoic acid
FKBP51: FK506 binding protein 51
GFAP: Glial fibrillary acidic protein
HDHA: Hydroxydocosahexaenoic acid
HEPE: Hydroxyeicosapentaenoic acid
HETE: Hydroxyeicosatetraenoic acid
HETrE: Hydroxyeicosatrienoic acid
HHTrE: Hydroxyheptadecatrienoic acid
HODE: Hydroxyoctadecadienoic acid
HOTrE: Hydroxyoctadecatrienoic acid
IBA1: Ionized calcium-binding adaptor molecule 1
IL: Interleukin
LA: Linoleic acid
LC–HRMS: Liquid chromatography–high-resolution mass spectrometry
PG: Prostaglandin
PLA_2_: Phospholipase A2
PUFA: Polyunsaturated fatty acids
SAFit2: Selective antagonist of FKBP51 by induced fit
SC: Spinal cord
TRP: Transient receptor potential
TRPV1: Transient receptor potential vanilloid 1 channel
TXB_2_: Thromboxane 2
