# Supplementary material for: SAFit2 ameliorates paclitaxel-induced neuropathic pain by reducing spinal gliosis and elevating pro-resolving lipid mediators
Source: J Neuroinflammation. 2023 Jun 24;20:149. doi: 10.1186/s12974-023-02835-5 (PMC10290418; doi:10.1186/s12974-023-02835-5)
Supplement: Supplementary file 1 — Additional file 1: Figure S1. Enhanced FKBP51 expression in spinal cord and L4-L5 DRG slices of paclitaxel-treated animals. Figure S2. Pharmacokinetic study of SAFit2 comparing two formulations. Figure S3. Reduced ATF3 expression in L4 and L5 DRGs of SAFit2-treated animals. Figure S4. Reduced cFOS expression in spinal cord and L4-L5 DRG slices of SAFit2-treated animals. Figure S5. Cytokines and chemokines measured in the DRGs of paclitaxel-treated mice. Figure S6. Cytokines measured in the spinal cord of paclitaxel-treated mice. Figure S7. Chemokines measured in the spinal cord of paclitaxel-treated mice. [file 12974_2023_2835_MOESM1_ESM.docx]

**Additional information**

**SAFit2 ameliorates paclitaxel-induced neuropathic pain by reducing spinal gliosis and elevating pro-resolving lipid mediators**

Saskia Wedel^1^, Lisa Hahnefeld^1,2^, Yannick Schreiber^2^, Christian Namendorf^3^, Tim Heymann^4^, Manfred Uhr^3^, Mathias V. Schmidt^3^, Natasja de Bruin^2^, Felix Hausch^4^, Dominique Thomas^1,2^, Gerd Geisslinger^1,2^ and Marco Sisignano^1,2*^

^1^ Institute of Clinical Pharmacology, *pharmazentrum frankfurt*/ZAFES, University Hospital, Goethe-University, 60590 Frankfurt am Main, Germany

^2^ Fraunhofer Institute for Translational Medicine and Pharmacology ITMP and Fraunhofer Cluster of Excellence for Immune Mediated Diseases CIMD, 60596 Frankfurt am Main, Germany

^3^ Core Unit Analytics and Mass Spectrometry, Max Planck Institute of Psychiatry, 80804 Munich, Germany

^4^ Department of Biochemistry, Technical University of Darmstadt, 64287 Darmstadt, Germany

*Address correspondence to: Dr. Marco Sisignano, *pharmazentrum frankfurt*/ZAFES, Institute of Clinical Pharmacology, University Hospital, Goethe-University, D-60590 Frankfurt am Main, Germany, Phone: +49 (0)69-6301-7819, E-mail: Marco.Sisignano@med.uni-frankfurt.de

Additional Figures: 7

**
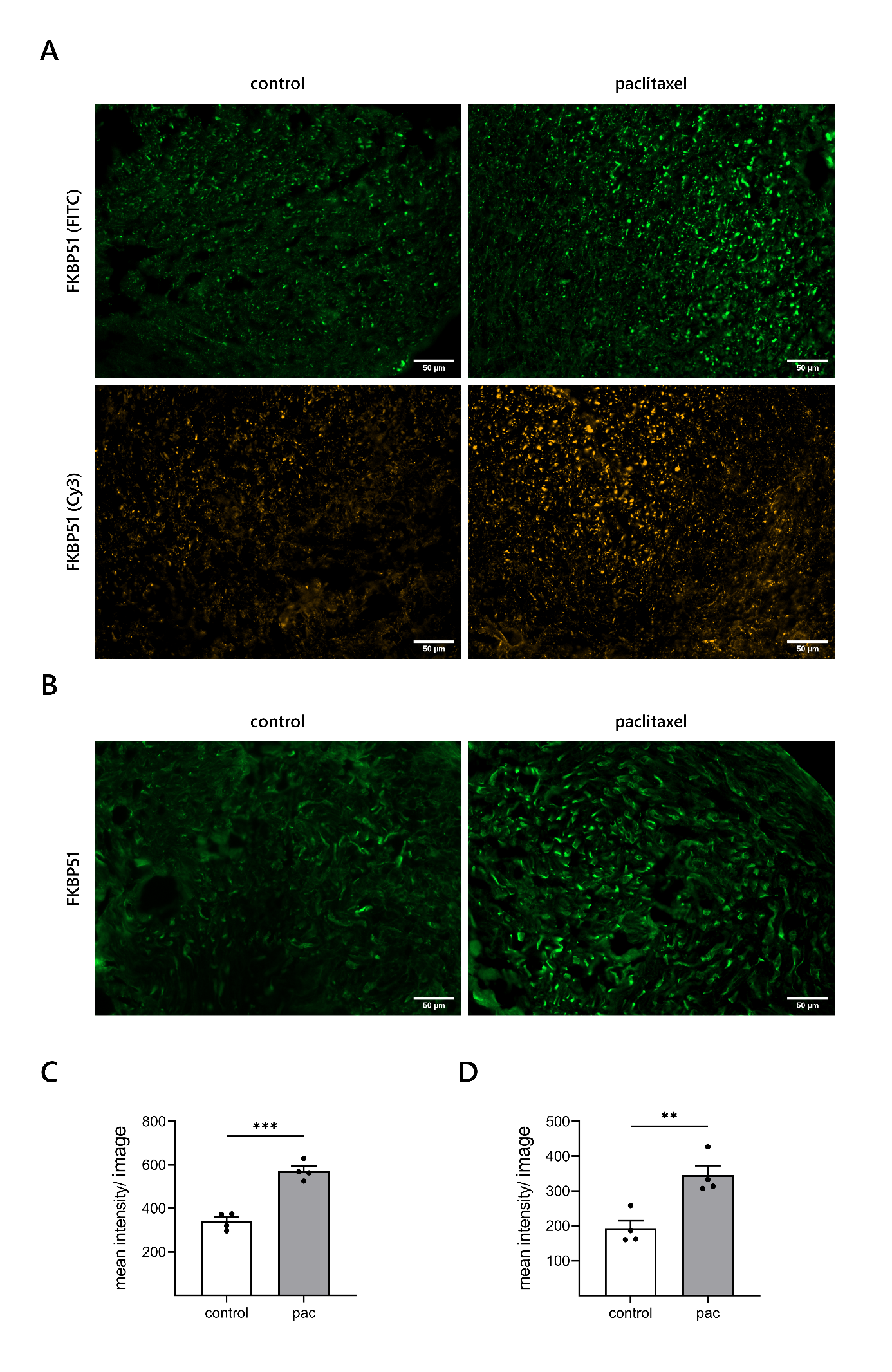
**

**Figure S1: Enhanced FKBP51 expression in spinal cord and L4-L5 DRG slices of paclitaxel-treated animals.** Immunohistochemistry staining of the protein FKBP51. Representative images of FKBP51 stained spinal cord (dorsal horn) **(A)** and L4-L5 dorsal root ganglia (DRG) **(B)** slices at 20X magnification (scale bar: 50 µm). Samples of naïve animals were labeled as control. Quantification of the mean intensity per image for spinal cord samples **(C)** and DRG samples **(D)**. Data represent the mean ± SEM from 10 quantified images per mouse. Each condition comprises data from four mice. ** p < 0.01, *** p < 0.001, student´s t-test with Welch´s correction.


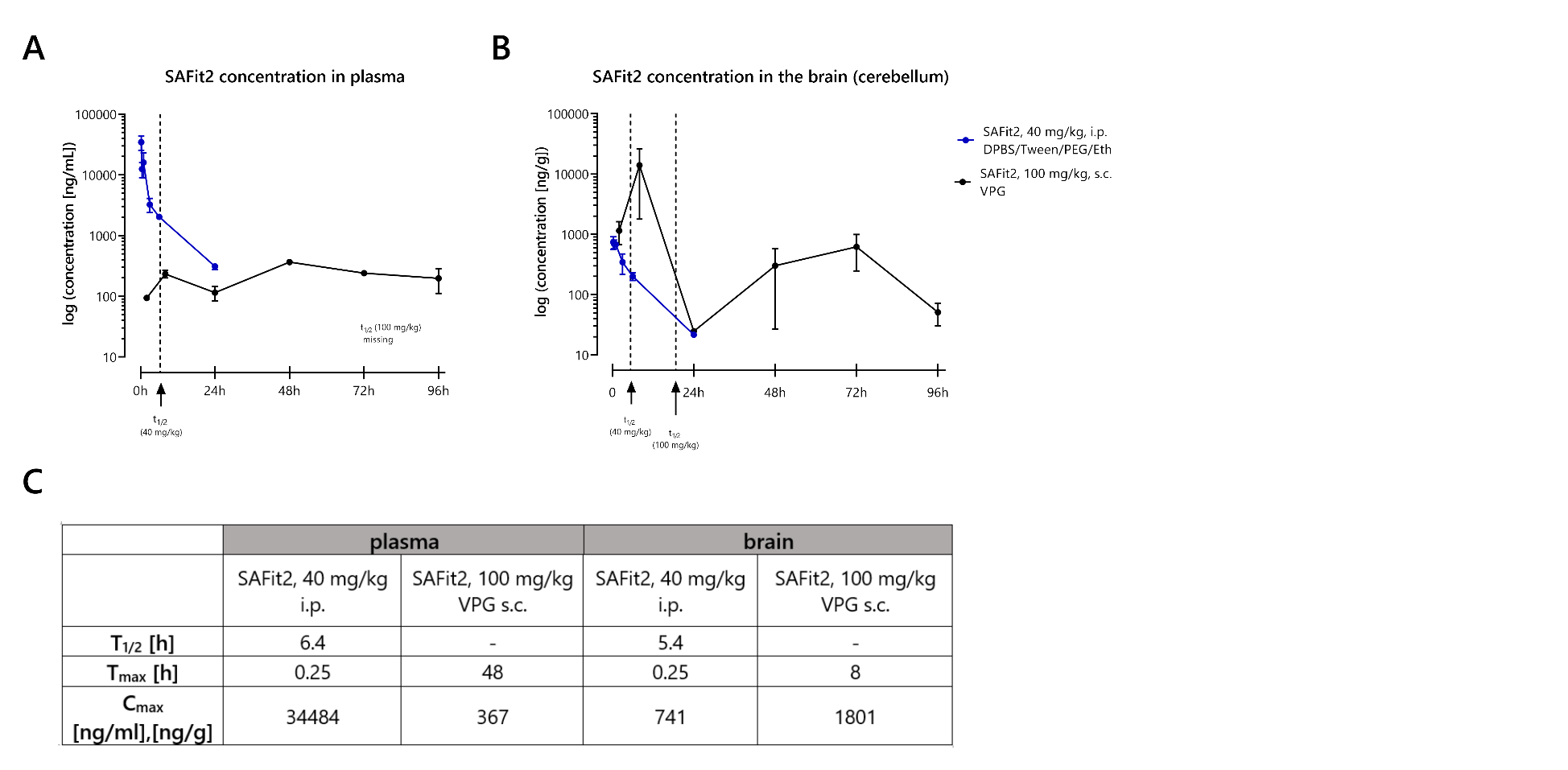


Figure S2: Pharmacokinetic study of SAFit2 comparing two formulations. The animals received one dose of SAFit2 formulated either in PBS supplemented with 5% PEG400, 5% Tween and 0.7% ethanol or as slow-release formulation in vesicular phospholipid gel (VPG). To assess the concentrations of SAFit2 in plasma (A) and brain (B), samples were collected from three mice for each indicated time point. (C) The table shows the calculated half-time as well as the maximum time and the maximum measured concentration of SAFit2.


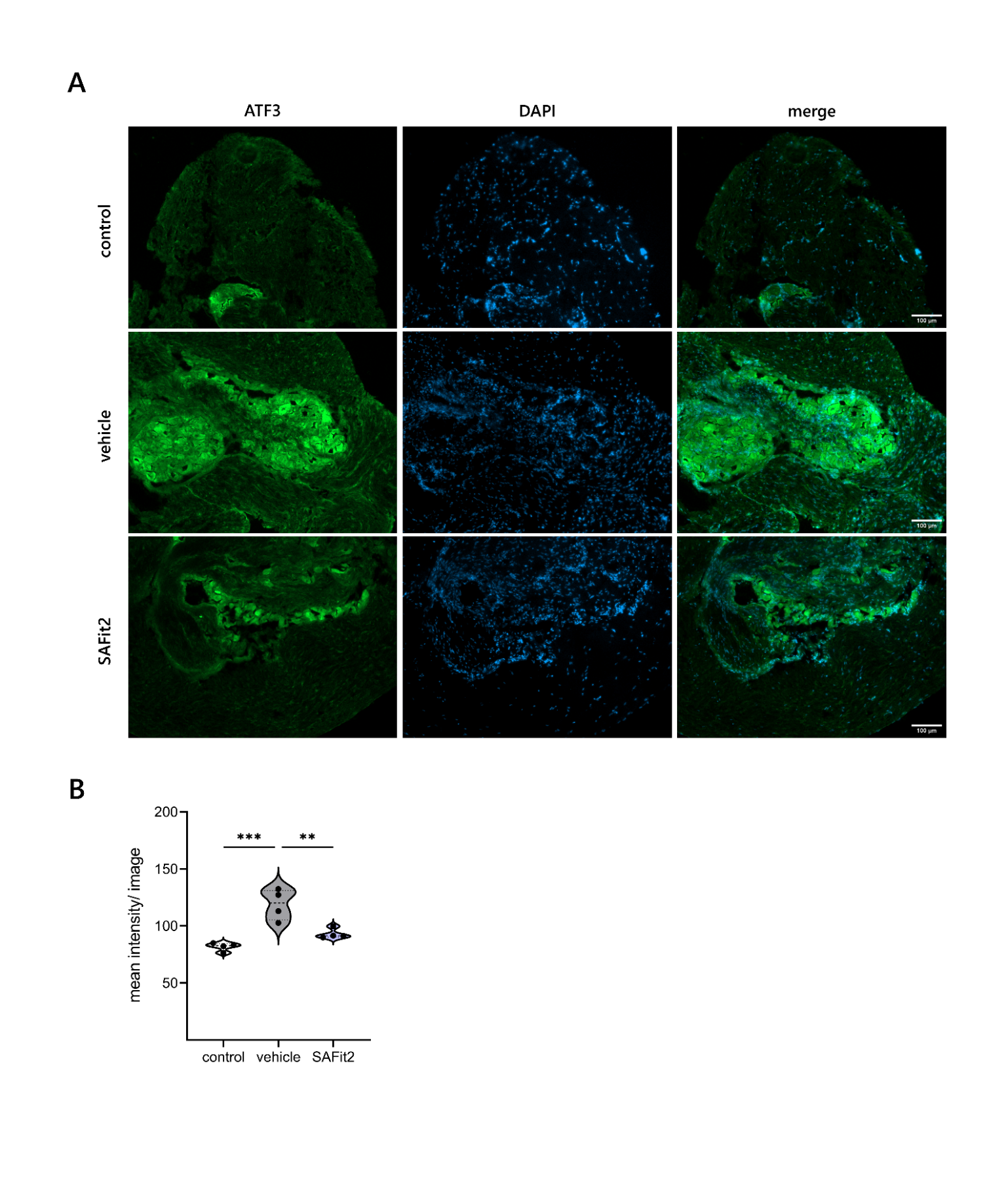


**Figure S3:** **Reduced ATF3 expression in L4 and L5 DRGs of SAFit2-treated animals.** Immunohistochemistry staining of the neuronal stress marker ATF3. **(A)** Representative images of ATF3 stained L4 and L5 dorsal root ganglia (DRGs) at 10X magnification (scale bar: 100 µm). Samples of naïve animals were labeled as control. **(B)** Quantification of the mean intensity per image. Data represent the mean ± SEM from 10 quantified images per mouse. Each condition comprises data from four mice. ** p < 0.01, *** p < 0.001 one-way ANOVA with Tukey´s multiple comparison test.

**
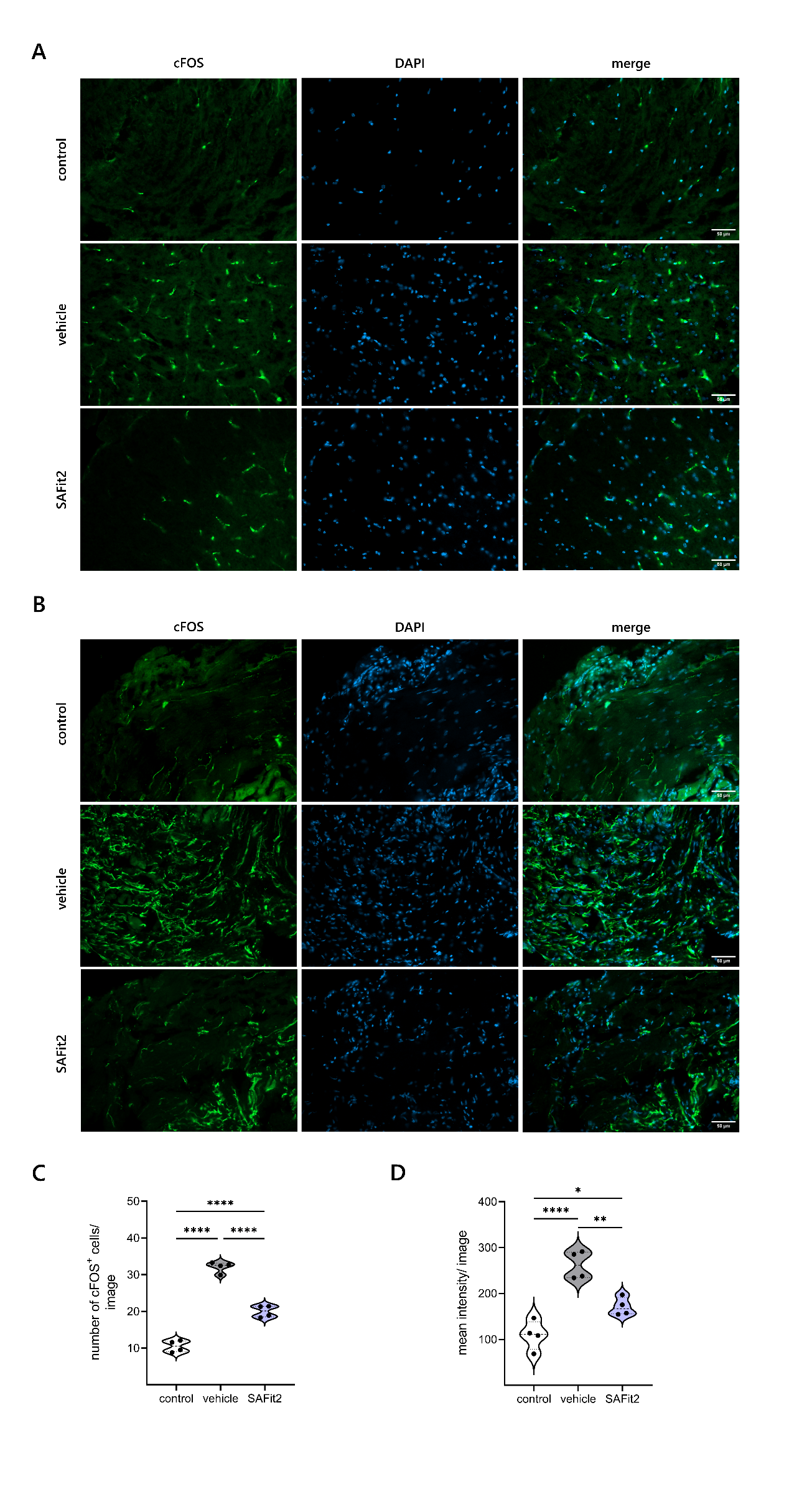
**

**Figure S4: Reduced cFOS expression in spinal cord and L4-L5 DRG slices of SAFit2-treated animals.** Immunohistochemistry staining of the neuronal activity marker cFOS. Representative images of cFOS stained spinal cord (dorsal horn **(A)** and L4-L5 dorsal root ganglia (DRGs) **(B)** slices at 20X magnification (scale bar: 50 µm). Samples of naïve animals were labeled as control. **(C)** Quantification of cFOS positive signals per image. **(D)** Quantification of the mean intensity per image. Data represent the mean ± SEM from 10 quantified images per mouse. Each condition comprises data from four mice. ** p < 0.01, *** p < 0.001, **** p < 0.0001 one-way ANOVA with Tukey´s multiple comparison test.


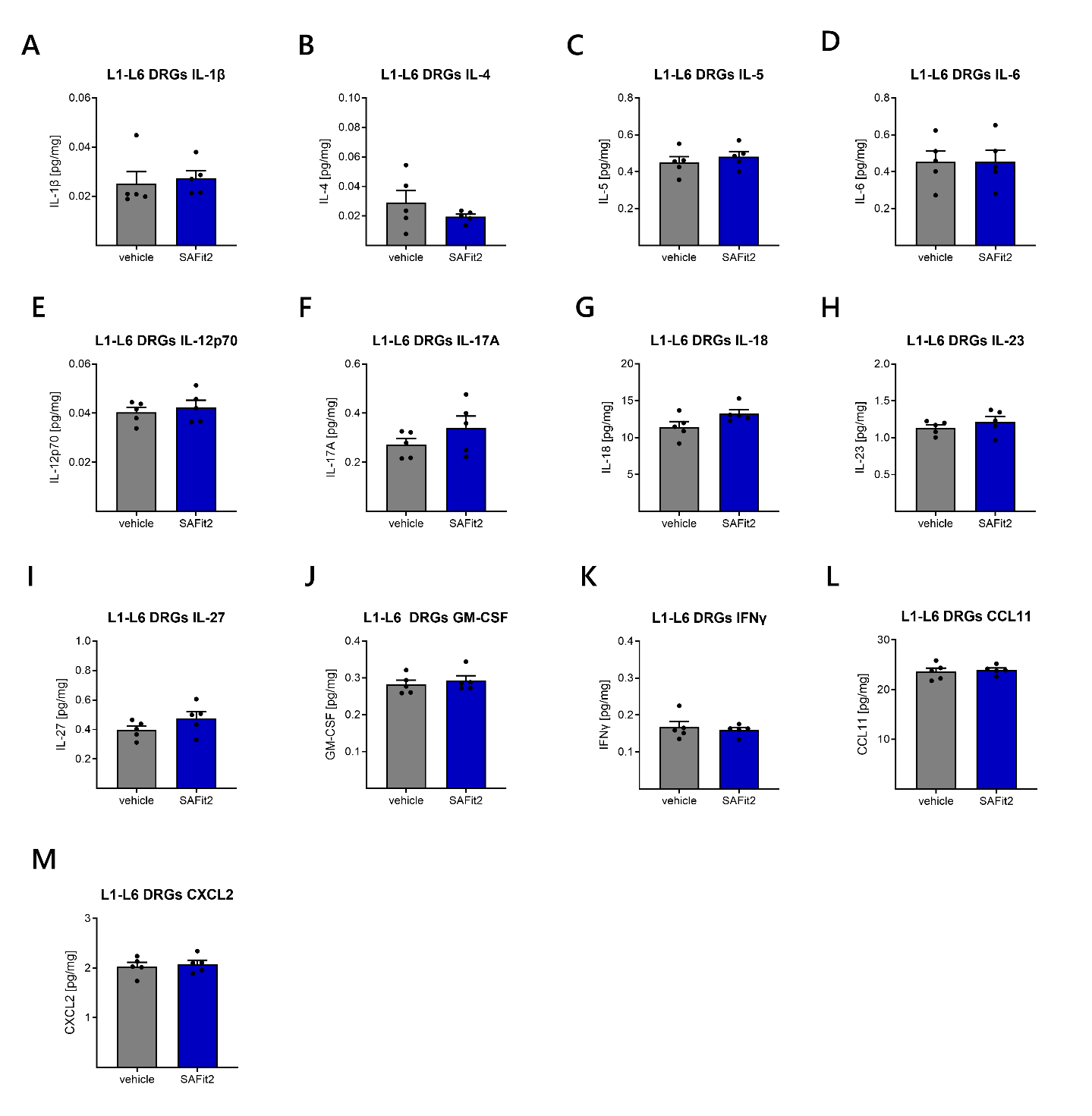


Figure S5: Cytokines and chemokines measured in the DRGs of paclitaxel treated mice. After 12 days, DRGs samples were homogenized and analyzed using a multiplex immunoassay including a panel of 26 cytokines and chemokines. The data represents the mean ± SEM from 5 mice per group. The raw data was related to the total protein amount of the sample.


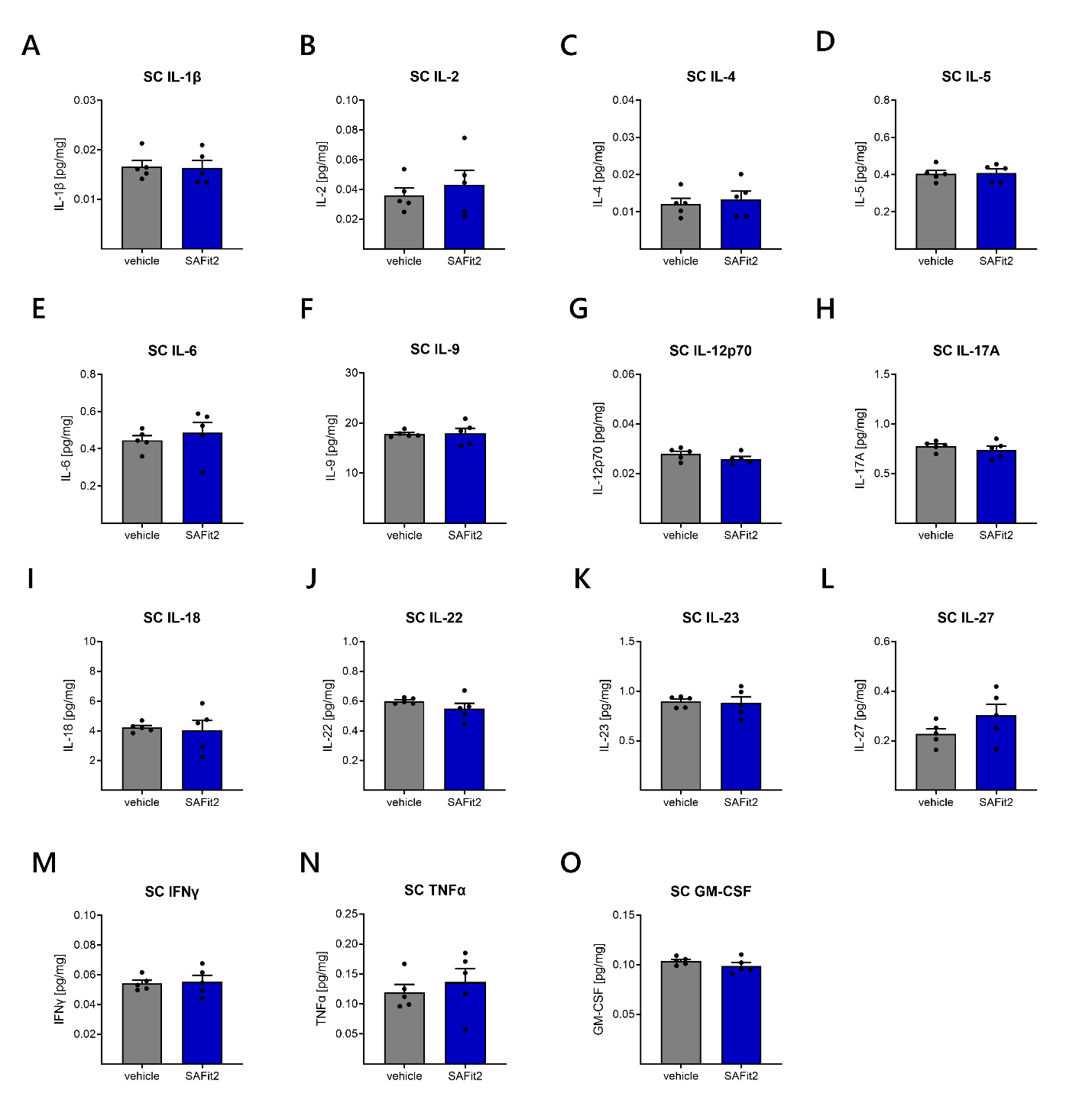
Figure S6: Cytokines measured in the spinal cord of paclitaxel treated mice. After 12 days, spinal cord samples were homogenized and analyzed using a multiplex immunoassay including a panel of 26 cytokines and chemokines. The data represents the mean ± SEM from 5 mice per group. The raw data was related to the total protein amount of the sample.


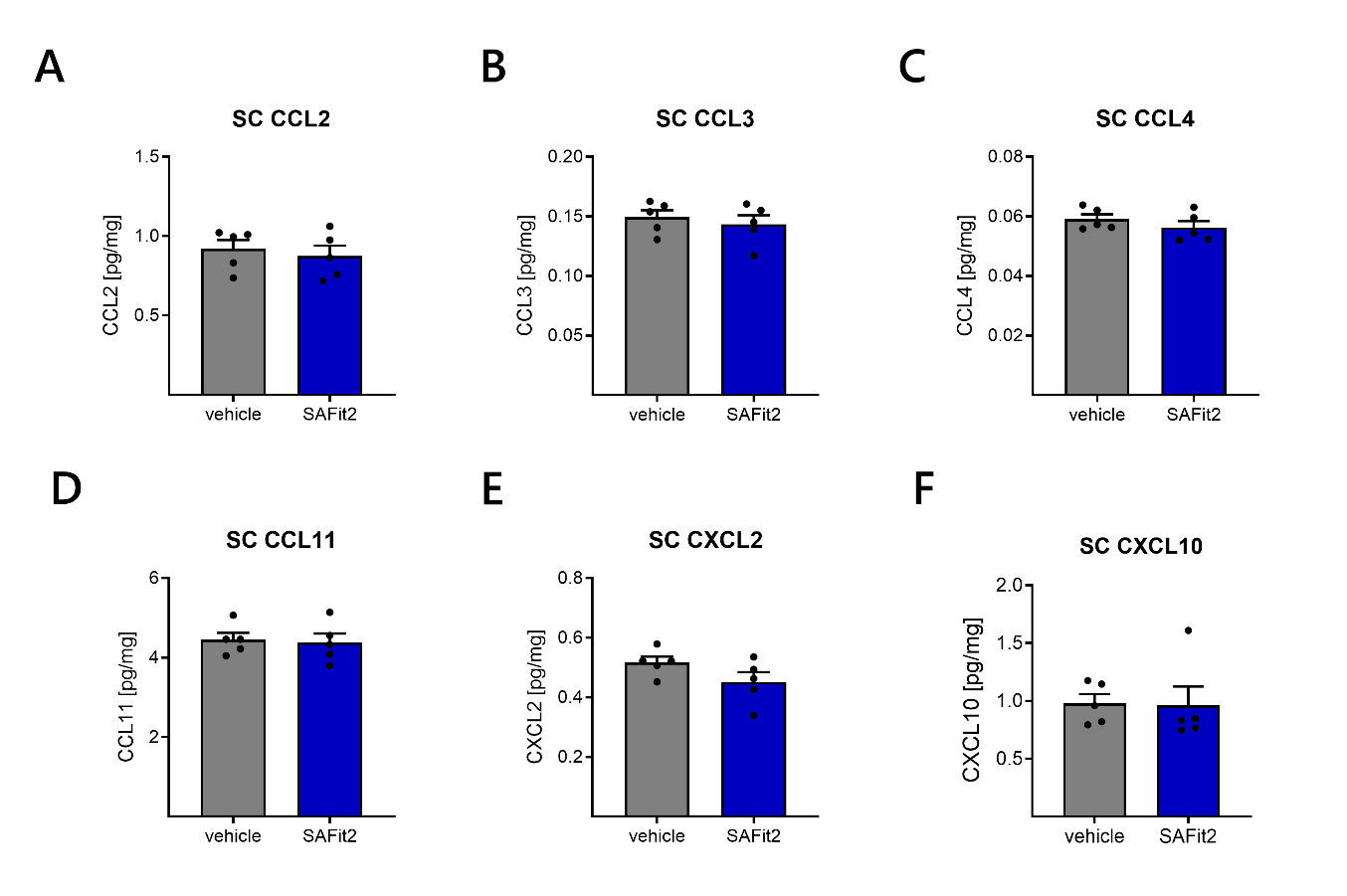


Figure S7: Chemokines measured in the spinal cord of paclitaxel treated mice. After 12 days, spinal cord samples were homogenized and analyzed using a multiplex immunoassay including a panel of 26 cytokines and chemokines. The data represents the mean ± SEM from 5 mice per group. The raw data was related to the total protein amount of the sample.

**Supplementary Methods section: DRG immunohistochemistry**

The L4 and L5 DRG samples were obtained as described in the “tissue isolation” method section in the main manuscript. Afterwards, the tissue samples were embedded in Tissue-Tek and frozen at -80 °C. For immunohistochemistry stainings, the frozen tissue samples were serially sliced into 12 µm tissue slices with a cryostat (Leica Biosystems). Next, the slices were stained according to the protocol, which is described in the ”immunohistochemistry” method section in the main manuscript. The primary antibodies anti-ATF3 (sc-188, Santa Cruz) and anti-cFOS (9F3, Cell Signaling) were diluted 1:50 and 1:200, as recommended, respectively in 1% BSA solution and applied for an overnight incubation at 4 °C. The primary antibody anti-FKBP51 (sc-271547, Santa Cruz) was diluted 1:50 as recommended in 3% BSA solution to minimize cross-reactivity as the host species is mouse. The secondary antibody goat anti-rabbit Alexa Fluor 488 (ab150077, Abcam) was diluted 1:1000 in 1% BSA solution and applied for one hour at room temperature. The secondary antibodies goat anti-mouse Alexa Fluor 488 (CF488A, Biotium) and sheep anti-mouse Cy3 (C2181, Sigma Aldrich) were diluted in 3% BSA solution. For quantification purposes, 10 images were taken per animal per staining. One treatment group comprises samples from four animals as for the spinal cord samples.
